# Virtual Screening of a Marine Natural Product Database for In Silico Identification of a Potential Acetylcholinesterase Inhibitor

**DOI:** 10.3390/life13061298

**Published:** 2023-05-31

**Authors:** Anushree Chandrashekhar Gade, Manikanta Murahari, Parasuraman Pavadai, Maushmi Shailesh Kumar

**Affiliations:** 1 Somaiya Institute of Research and Consultancy, Somaiya Vidyavihar University, Mumbai 400077, India; 2 Department of Pharmacy, Koneru Lakshmaiah Education Foundation, Vaddeswaram 522302, India; 3 Department of Pharmaceutical Chemistry, Faculty of Pharmacy, M.S. Ramaiah University of Applied Sciences, Bengaluru 560054, India

**Keywords:** Alzheimer’s disease, marine natural products, acetylcholinesterase inhibitors, molecular docking, MD simulations

## Abstract

Alzheimer’s disease is characterized by amyloid-beta aggregation and neurofibrillary tangles. Acetylcholinesterase (AChE) hydrolyses acetylcholine and induces amyloid-beta aggregation. Acetylcholinesterase inhibitors (AChEI) inhibit this aggregation by binding to AChE, making it a potential target for the treatment of AD. In this study, we have focused on the identification of potent and safe AChEI from the Comprehensive Marine Natural Product Database (CMNPD) using computational tools. For the screening of CMNPD, a structure-based pharmacophore model was generated using a structure of AChE complexed with the co-crystallized ligand galantamine (PDB ID: 4EY6). The 330 molecules that passed through the pharmacophore filter were retrieved, their drug-likeness was determined, and they were then subjected to molecular docking studies. The top ten molecules were selected depending upon their docking score and were submitted for toxicity profiling. Based on these studies, molecule 64 (CMNPD8714) was found to be the safest and was subjected to molecular dynamics simulations and density functional theory calculations. This molecule showed stable hydrogen bonding and stacked interactions with TYR341, mediated through a water bridge. In silico results can be correlated with in vitro studies for checking its activity and safety in the future.

## 1. Introduction

Dementia refers to a health condition which causes deterioration in cognitive functions, impacting memory, thinking, reasoning skills, and the performance of daily activities. Dementia mainly affects elderly people. According to the World Health Organization (WHO), 55.2 million people are currently living with dementia. Alzheimer’s disease (AD) is the most common form of dementia, contributing to around 60–70% of cases [1]. The disease is characterized by a progressive decline in cognition and memory, and behavioral manifestations. According to the National Institute of Aging (NIA), AD symptoms include memory loss, poor judgement, loss of spontaneity, wandering and getting lost, misplacing or losing things, increased anxiety and aggression, withdrawal from social activities, difficulty in carrying out familiar tasks, etc. [2]. The important pathological hallmarks of AD include accumulation of amyloid plaques, formation of neurofibrillary tangles, loss of neuronal and synaptic functions, astrogliosis, and microglial cell activation [3].

AD treatments are broadly classified depending upon their nature, i.e., disease-modifying therapies and symptomatic therapies. Drug targets for disease-modifying therapies include (A) modulation of Aβ (amyloid-beta) production via alpha-secretase enhancement, beta-secretase inhibition, gamma-secretase inhibition, and gamma-secretase modulation; (B) Aβ degradation via neprilysin activation and insulin-degrading enzyme activation; (C) Aβ removal via receptors, vaccination, passive immunization and prevention of entry from periphery; (D) prevention of Aβ toxicity via prevention of aggregation by binding to Aβ and prevention of oligomerization of amyloid protein; and (E) prevention of tau aggregation and hyperphosphorylation [4]. Recently, the United States-Food and Drug Administration (US-FDA) has approved two drugs (monoclonal antibodies)—Aducanumab and Lecanemab—for the treatment for AD, which act as disease-modifying agents by reducing Aβ plaques in the brain and ultimately slow down disease progression [5,6]. On the contrary, drug targets for symptomatic treatments for AD include (A) acetylcholinesterase (AChE) inhibition, (B) N-methyl-D-aspartate (NMDA) receptor modulation, (C) nicotinic acetylcholine receptor activation, (D) gamma-aminobutyric acid receptor blockade, (E) serotonin receptor activation and blockade, (F) histamine H3 receptor blockade, and (G) phosphodiesterase inhibition [4]. In order to treat the symptoms of AD, donepezil, galantamine, rivastigmine, and memantine have been approved by the US-FDA and are currently in use [7]. AD-35 is another potential drug candidate under clinical trials, exhibiting acetylcholinesterase inhibition [8].

A comparison of disease-modifying therapies and symptomatic treatments shows that the failure rate of disease-modifying agents is comparatively high. Reasons underlying this include insufficient understanding of AD pathophysiology, inappropriate drug doses, and unresponsiveness towards monotherapy treatments owing to the complex pathophysiology of AD. Several examples of disease-modifying agents that failed in phase 3 clinical trials are Semagecestat and Avagacestat (gamma-secretase inhibitors); Lanabecestat, Verubecestat, and Atabecestat (BACE1 inhibitors); and Bapinezumab and Solanezumab (monoclonal antibodies). Some of these agents were reported to worsen daily functioning and even increased the rate of progression of disease [9]. Hence, the search for novel therapies to prevent and treat Alzheimer’s is gaining importance.

Acetylcholine (ACh) is the most consistent neurotransmitter secreted by cholinergic neurons and plays a major role in neurotransmission involved in memory and learning. The main function of AChE is the breakdown of ACh at cholinergic synapses [10]. Some evidence suggests a role for AChE in the formation of Aβ aggregates. Aβ is a 28–43-residue peptide fragment derived from a highly hydrophobic domain of amyloid precursor protein (APP). AChE is reported to form a stable complex with Aβ plaques and hence researchers have suggested its involvement in the induction of fibril formation [11]. In addition to this, as per the cholinergic hypothesis, AD is marked by loss of cholinergic neurons, and alterations in ACh-synthesizing enzyme choline acetyltransferase (ChAT) and AChE. This loss results in downregulation of acetylcholine levels leading to decreased cholinergic neurotransmission. Owing to its important role in the regulation of cholinergic neurotransmission and the induction of Aβ aggregation, AChE is one of the viable targets for the improvement in AD symptoms. Treatment with acetylcholinesterase inhibitors (AChEI) in AD increases the concentration of acetylcholine in the brain by reversing the cholinergic deficit, eventually resulting in increased signal transduction and improved cholinergic function. They also help in delaying memory loss and managing day-to-day activities [10,12]. Aβ aggregation is also supposed to be suppressed by treatment with AChEI [13]. 

Currently, available drug treatments for mild to moderate AD which target AChE exert several side-effects, such as nausea, diarrhoea, dizziness, weight loss, abdominal pain, anorexia, and vomiting [14]. Hence, researchers are looking for more effective drugs with maximum efficacy and minimum side effects. In this experiment we have mainly focused on the inhibition of AChE-induced Aβ aggregation by targeting AChE. Natural compounds possessing AChE-inhibiting potential can also be used as AChEI, owing to their safety as compared to synthetic molecules. One such example is galantamine, obtained from *Galanthus woronowii*. Galantamine was approved by the US-FDA in 2001 and is currently used for the treatment of AD [15]. Many studies are nowadays performed to check the potential of natural compounds such as rutin, quercetin, etc., for the treatment of AD [16,17]. 

**Figure 1:**
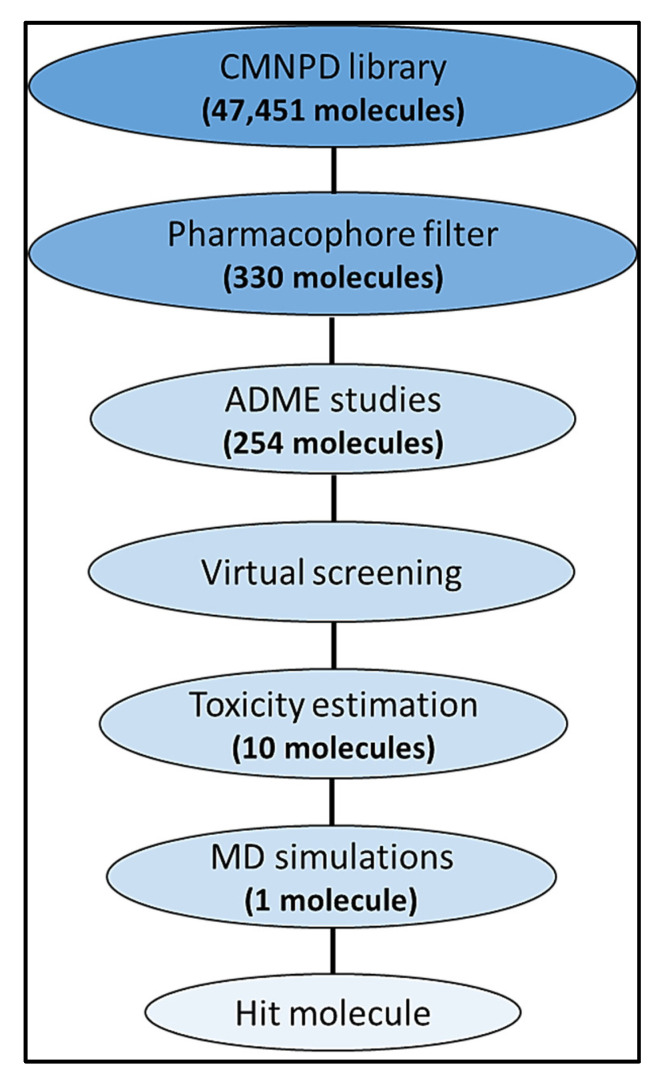
Steps followed in the virtual screening of the natural products database.

Molecular docking is a computational drug design approach widely employed in lead discovery because it reduces both the time and cost of the discovery process. In this approach, ligand–receptor interactions are studied with the help of computer software. The docking system is based on scoring functions that indicate the binding energy of the ligands when bound to their receptors. Our work aims to perform a virtual screening of the Comprehensive Marine Natural Product Database (CMNPD) against AChE binding sites. Several marine natural products are reported to possess various biological properties. These marine natural product sources are typically marine bacteria, fungi, fish, seaweed, shellfish, etc. (https://www.cmnpd.org/, accessed on 10 February 2023) [18]. A pharmacophore model was built with the help of the Pharmit server, employing a structure of AChE (PDB ID: 4EY6) complexed with galantamine (GNT) as an input for the virtual screening of the 47,451 molecules contained in the CMNPD library. The library was screened against the generated structure-based pharmacophore model. The drug-likeness of the filtered molecules was evaluated using SwissADME, followed by molecular docking of the drug-like molecules using AutoDock Tools 1.5.6 (AutoDock Vina, Biovia Discovery Studio Visualizer). The top ten molecules possessing the lowest docking scores were determined and their toxicity was tested virtually using ProTox-II. A molecular dynamics (MD) study of the molecule with the highest safety and efficacy was performed using the Desmond tool from Schrodinger. This study suggests a potential use of computational approaches when searching for and designing potent and safe drugs. The steps followed in the virtual screening for the discovery of novel molecules are depicted in Figure 1.

## 2. Materials and Methods

### 2.1. Dataset of Marine Natural Compounds

The chemical structures of the marine natural compounds were obtained from CMNPD, (https://www.cmnpd.org/cmnpd/supplement/Downloads/CMNPD_1.0_3d.sdf.gz, accessed on 10 February 2023). The complete library of 47,451 molecules was screened to search for novel AChEI.

### 2.2. Structure-Based Pharmacophore Modelling and Virtual Screening

The 3D pharmacophore search was carried out with the help of the Pharmit server (https://pharmit.csb.pitt.edu/, accessed on 13 February 2023) [19]. The pharmacophore model was generated by loading the structure of AChE (PDB ID: 4EY6) complexed with a GNT ligand (galantamine) as an input. The Pharmit parameters for the 3D pharmacophore search remained unchanged. Virtual screening of the complete library was carried out and a dataset of selected compounds was retrieved. In addition to this, molecules were also filtered using an additional filter of RMSD ≤ 2 A° for hit screening. Based on the generated pharmacophore model, the CMNPD library containing 47,451 molecules was screened. A complete list of 330 molecules was obtained from the Pharmit server that passed through the pharmacophore filter.

### 2.3. Structure Preparation

The 3D crystal structure of AChE in complex with an inhibitor, galantamine (PDB ID: 4EY6), was downloaded in PDB format from the Protein Data Bank (https://www.rcsb.org/, accessed on 13 February 2023) and was prepared using Biovia Discovery Studio Visualizer. During protein preparation, heteroatoms, water molecules, and unwanted ligand atoms were deleted. Polar hydrogen atoms were added to heavy atoms. Charges were added and evenly distributed throughout the structure. The 3D structures of all the selected ligand molecules, as well as the standard drug, were prepared for docking using Biovia Discovery Studio Visualizer and AutoDock Vina software.

### 2.4. Prediction of Drug-Likeness Features

ADME (absorption, distribution, metabolism, excretion) parameters constitute an important part of safety and efficacy analyses of a molecule inside the body. Considerable and adequate absorption is a distinctive characteristic of any drug substance given orally. Lipophilicity and hydrophilicity of the molecule should be well-balanced, as both features impact the absorption and distribution of the molecule inside the body. Similarly, efficacy and toxicity are also vital considerations for any therapy. Hence, after the metabolism of any drug, the properties of its metabolites should be known, and it should be eliminated from the body after its therapeutic effect is obtained. All of the molecules that passed through the pharmacophore model were screened to evaluate their drug-likeness using SwissADME, a free online tool available at (http://www.swissadme.ch/, accessed on 27 February 2023), which enables the easy determination of the pharmacokinetics and drug-likeness of molecules [20,21].

### 2.5. Molecular Docking

Drug-like molecules screened from SwissADME were subjected to molecular docking studies. The aim of these studies was to evaluate the interactions of amino acid residues in the active pocket, and the docking scores of ligands with the selected target protein. Docking experiments were performed using AutoDock Vina software. The grid box dimensions were recorded using Biovia Discovery Studio Visualizer, that covered the active site of the target protein to a minimum specific area and aided site-specific docking of the ligand. This active site was defined using the co-crystallized ligand present in the protein structure. The interactions between the ligands and the receptor were recorded in terms of the docking score. After completion of docking, the docked outputs of all the ligands were screened to identify the top ten molecules with the lowest docking scores. The best binding poses with the lowest docking scores were utilized for further investigation of interacting residues and binding interactions between the ligands and the receptor.

### 2.6. Toxicity Estimation

The toxicity of the top ten molecules (in terms of docking scores and drug-likeness properties) was predicted using the ProTox-II website (https://tox-new.charite.de/protox_II/, accessed on 20 March 2023) [22]. Oral toxicity (mg/kg) values and the toxicity classes of the molecules were predicted. 

### 2.7. Molecular Dynamics Simulations

Based on the docking score, binding interactions in the active pocket of the target protein and ADMET calculations, molecule 64 (CMNPD8741) was selected for MD simulations for 200 ns. In addition, the standard drug donepezil was submitted for MD simulations, using the Desmond tool from Schrodinger. Docked poses of the respective complexes obtained from AutoDock were loaded into the interface of Desmond and the TIP3P-type water model box was selected. The OPLS-AA force field was applied for running the simulation. In addition, the Reversible Reference System Propagator Algorithm (RESPA) was employed to handle short time steps for fast forces such as bonded interactions, and longer time steps for slow forces such as non-bonded interactions. The system was neutralized by adding counter-ions and submitted for the minimization process. Respective minimized complexes were submitted to MD simulations for 200 ns at 300 K temperature and 1.013 bar pressure conditions. The Langevin dynamics algorithm was also utilized to control the temperature with the help of the Langevin damping coefficient parameter. Similarly, pressure was regulated by the Martyna-Tobias-Klein (MTK) method. The particle Mesh Ewald (PME) method accounted for electrostatic interactions. After completion of the simulation, the entire trajectory was analyzed to retrieve the RMSD plots, RMSF plots, and hydrogen bonding, hydrophobic, water and salt bridge, and 2D interaction plots of the respective complexes.

### 2.8. Density Functional Theory

The electronic properties of a molecule are essential for its therapeutic benefits. Density Functional Theory (DFT) calculations are effective at measuring the electronic properties in a 3D system, applying the fundamental laws of quantum mechanics. Here, hit molecules obtained from preliminary calculations and the standard drug donepezil were considered using the Gaussian 03W program and the Gauss View molecular visualization tools. The molecular structures of both compounds were optimized by the DFT/Becke-3–Lee–Yang–Parr (B3LYP) technique under the 6-311G (d, p) basis set. The EHOMO (molecular orbital energy of the highest occupied molecular orbital) and ELUMO (molecular orbital energy of the lowest unoccupied molecular orbital) values in electron volts (ev) were recorded and the energy gap was calculated. In addition, energy diagrams were retrieved using the visualization tool, Gauss View. 

## 3. Results and Discussion

### 3.1. Structure-Based Pharmacophore Modelling

Pharmacophore models indicate all the important structural features taking part in drug–target (protein–ligand) interactions. These features include the spatial arrangement of the interacting atoms. Structure-based pharmacophore modelling employs the 3D structure of a target protein with its co-crystallized ligand to accurately identify the binding pocket of the receptor. To screen the CMNPD library, we used the crystal structure of recombinant human AChE complexed with galantamine (PDB ID: 4EY6).

**Figure 2:**
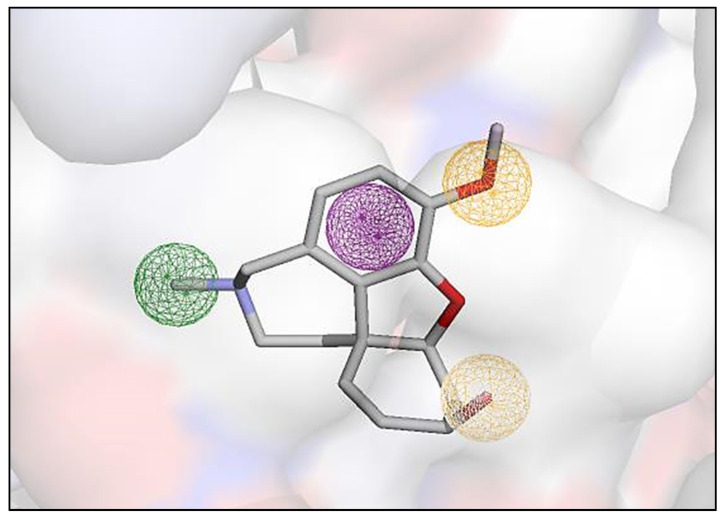
The pharmacophore model generated by the Pharmit server, including HBA (orange spheres), HBD (white sphere), the aromatic center (violet sphere), and the hydrophobic center (green sphere).

The pharmacophore model was generated using the Pharmit server. The pharmacophore features of ligands binding to the enzyme’s active site obtained from the server included two hydrogen bond acceptors, one hydrogen bond donor, one aromatic ring and one hydrophobic center at a specific distance. The screened molecules with similar pharmacophore features were retrieved with the help of query results obtained from the server. The generated pharmacophore hypothesis is depicted in Figure 2. This model aids in recognizing the structural requirements for the inhibition of AChE.

The proposed pharmacophore model was used to screen the CMNPD library (47,451 molecules). From this database, a set of 330 molecules was retrieved and subjected to drug-likeness estimation.

### 3.2. ADME Studies

The drug-likeness of the 330 molecules was evaluated using SwissADME. Any lead/hit molecule that is intended for use as a therapeutic agent must possess drug likeness and should also be safe. Drug-likeness is examined by passing the drug candidate through Lipinski’s rule of five, the Ghose filter, the Veber filter, the Egan filter, and the Muegge filter. Lipinski’s rule of five that sets criteria for drug-like properties states that the molecule should have a molecular weight ≤ 500 Da, log P ≤ 5, hydrogen bond donors (HBD) ≤ 5, and hydrogen bond acceptors (HBA) ≤ 10. The Ghose filter requires that the calculated log P should be between −0.4 and 5.6, the molecular weight should be between 160 and 480, the molar refractivity (MR) should be between 40 and 130, and that the total number of atoms should be between 20 and 70. The Veber filter includes that the number of rotatable bonds (nRB) ≤ 10 and that the total polar surface area (TPSA) ≤ 140 A°. Egan’s filter requires the following conditions: WLOGP (lipophilicity) ≤ 5.88 and TPSA ≤ 131 A°. Muegge’s filter requires the following conditions: 200 Da≤ molecular weight ≤ 600 Da, −2 ≤ XLOGP3 (lipophilicity) ≤ 5, TPSA ≤ 150 A°, number of rings ≤ 7, number of carbons > 4, number of heteroatoms > 1, nRB ≤ 15, HBA ≤ 10, and HBD ≤ 5 [23,24]. Other properties evaluated by SwissADME include GI absorption, BBB permeability, solubility, and solubility class. Molecules that violated the above-mentioned rules were excluded and SwissADME obtained 254 molecules from the data which possessed drug-like characteristics. The data obtained from SwissADME is represented in Table S1 in the Supplementary Materials.

### 3.3. Molecular Docking of Compounds from the CMNPD Database

**Figure 3:**
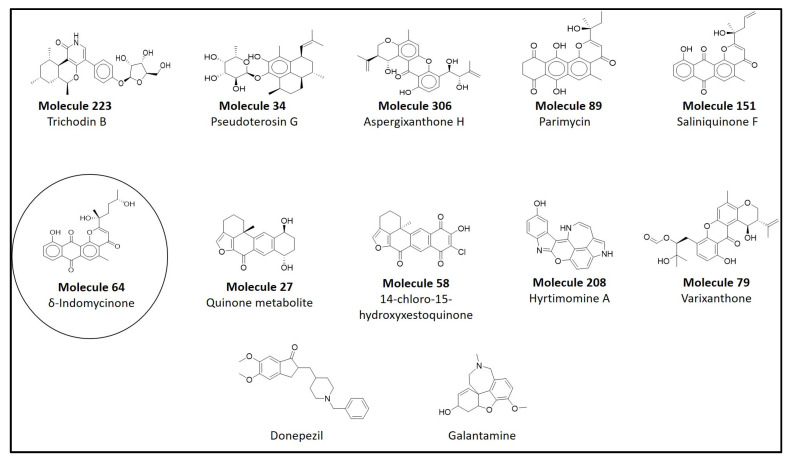
The names and chemical structures of in silico potential AChEI along with a standard AChEI. The chemical structure of the hit molecule (CMNPD8741) is encircled in the figure above.

Molecular docking is a powerful technique for the virtual screening of chemical databases and the selection of structures for molecular dynamics simulations. However, docking has its own limitations and previous studies reported that none of the docking tools could calculate the binding affinity towards the target [25,26]. Molecular docking is important in determining the binding interactions of molecules screened and retrieved from the CMNPD library. Docking studies of the drug-like molecules and an AChEI currently employed for the treatment of AD (donepezil) were carried out using AutoDock Vina. The 254 molecules screened using SwissADME were subjected to molecular docking with the target protein PDB ID: 4EY6. The molecular interactions and the docking scores of the selected compounds were compared with donepezil. Depending upon the docking score, the top ten compounds were selected as depicted in Figure 3. 

**Figure 4:**
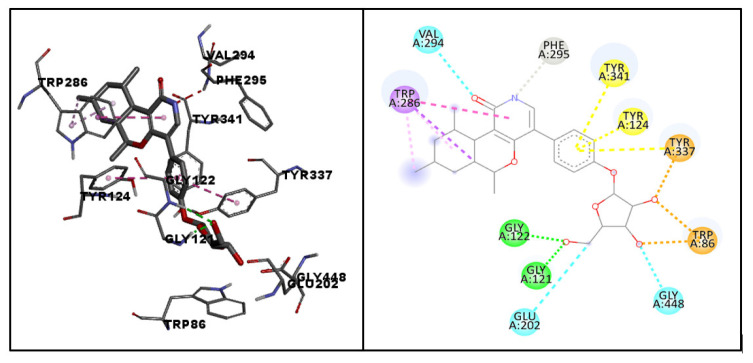
The 2D docking pose and binding interactions of CMNPD24838 with AChE (PDB: 4EY6). The dotted lines in various colors represent different types of interactions such as conventional hydrogen bonding (green), carbon hydrogen bonding (blue), pi–sigma interactions (violet), pi–pi T-shaped interactions (yellow), pi–donor hydrogen bonding (orange), donor–donor interactions (grey), pi–pi stacked interactions (dark pink) and pi–alkyl interactions (light pink).

**Figure 5:**
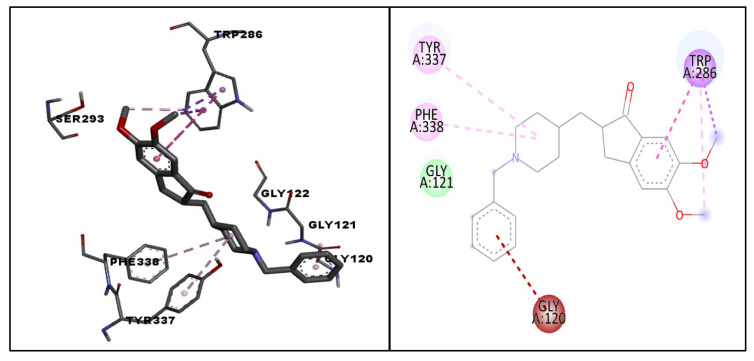
The docked pose and binding interactions of the standard drug donepezil with the target protein (PDB:4EY6). The dotted lines in various colors represent different types of interactions, such as pi–alkyl interactions (light pink), pi–sigma interactions (violet), pi–pi stacked interactions (pink), Van der Waal’s interactions (light green), and amide pi-stacked interactions (brown).

**Figure 6:**
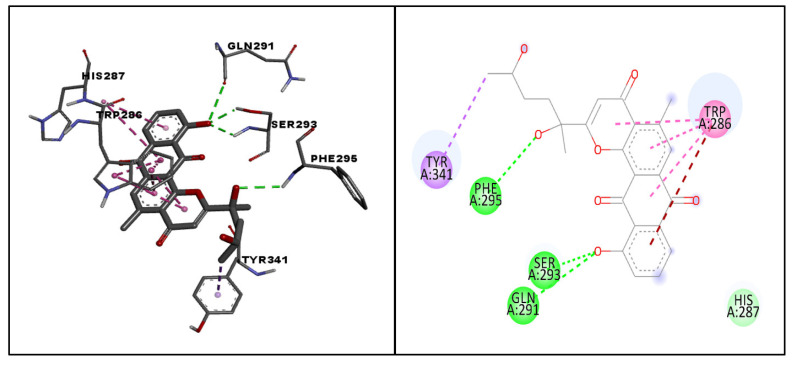
The docked pose and binding interactions of the standard drug galantamine with the target protein (PDB:4EY6). The dotted lines in various colors represent different types of interactions, such as conventional hydrogen bonding (green), carbon hydrogen bonding (blue), pi–alkyl interactions (light pink), pi–sigma interactions (violet), Van der Waal’s interactions (light green), and amide pi-stacked interactions (brown).

The docking scores of the top ten molecules ranged from −12.6 to −10.7 kcal/mol. The results obtained from molecular docking indicated that molecule 223 (CMNPD24838) exhibited the highest docking score of −12.6 kcal/mol and demonstrated conventional hydrogen bonding with GLY122 and GLY121; carbon hydrogen bonding with VAL294, GLY448, and GLY202; a donor–donor interaction with PHE295; pi–donor hydrogen bonding with TYR337 and TRP86; a pi–sigma interaction, a pi–pi stacked interaction, and a pi–alkyl interaction with TRP286; and pi–pi T-shaped interactions with TYR341 and TYR124 (Figure 4). Similarly, the binding interactions of donepezil and galantamine were also studied. The standard drug donepezil exhibited the following interactions with amino acid residues of the target protein: a Van der Waal’s interaction with GLY121; a pi–sigma interaction, a pi–pi stacked interaction and a pi–alkyl interaction with TRP286; an amide pi-stacked interaction with GLY120; and pi–alkyl interactions with TYR337 and PHE338 (Figure 5). Galantamine demonstrated conventional hydrogen bonding with GLU202; carbon hydrogen bonding with TYR337; a pi–sigma interaction with TRP86; an amide pi-stacked interaction with GLY121; pi–alkyl interactions with TRP286, PHE338, PHE297, PHE295, GLY122, and HIS447; and a Van der Waal’s interaction with GLY122 (Figure 6). The docking scores of the top ten molecules and the standard drug, along with their interacting residues, are summarized in Table 1 and depicted in the Supplementary Materials (Figures S1–S10). The ligand interactions with the target proteins were studied using AutoDock Vina and Biovia Discovery Studio Visualizer. 

### 3.4. Toxicity Estimation

Depending upon the docking scores of the molecules with the target protein, the top ten molecules with the lowest docking scores were selected for virtual toxicity estimation. ProTox-II was employed for the analysis of the oral toxicity LD_50_ value. The toxic doses and the toxicity classes of the molecules were predicted, and the data obtained from ProTox-II is depicted in Table 2. Molecule 64 exhibited the most negligible toxic dose of 4000 mg/kg body weight. This information is very important for hit identification because both the safety and efficacy of the drug molecules are of equal importance.

**Figure 7:**
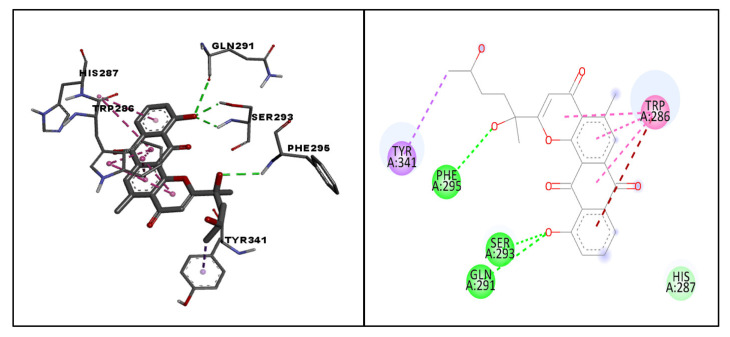
The docked pose and binding interactions of hit molecule (molecule 64) with the target protein (PDB:4EY6). The dotted lines in various colors represent different types of interactions such as hydrogen bonding (green), pi–sigma interactions (violet), pi–pi stacked interactions (pink), Van der Waal’s interactions (light green), and amide pi-stacked interactions (brown).

The docking pose and binding interactions of the hit molecule (molecule 64) with the target protein are depicted in Figure 7. The amino acid residues involved in the binding interactions of the hit molecule with the target protein were identified with the help of AutoDock Vina and Biovia Discovery Studio Visualizer. Molecule 64 exhibited conventional hydrogen bonding with PHE295, SER293 and GLN291; a pi–sigma interaction with TRP341; a pi–pi stacked interaction with TRP286; a Van der Waal’s interaction with HIS287; and an amide pi-stacked interaction with TRP286. Considering the above results, molecule 64, with maximum safety and a considerable docking score, was considered as a hit molecule and its MD studies were carried out.

### 3.5. Molecular Dynamics Simulations

**Figure 8:**
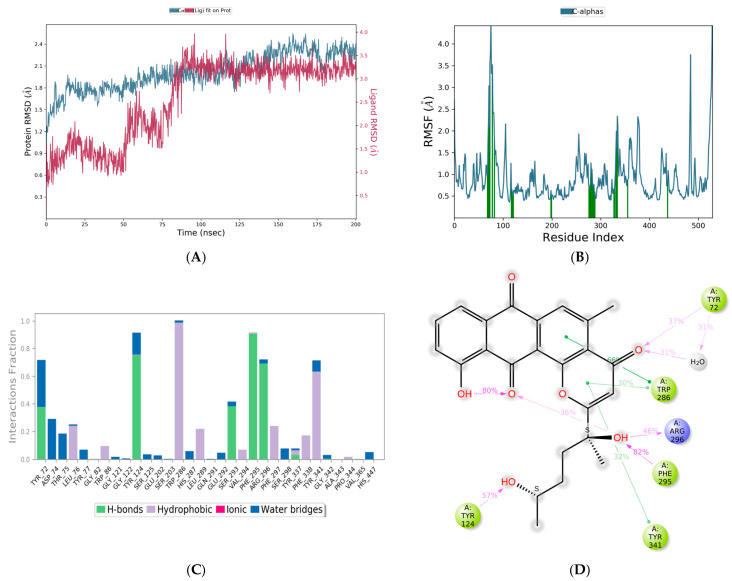
Molecular Dynamics Simulation plots of molecule 64 complexed with acetylcholinesterase (PDB ID: 4EY6). RMSD plot (**A**); RMSF plot (**B**); hydrogen bonding interactions, note for 8B: Green-Protein residues that interact with the ligand are marked with green-colored vertical bar (**C**); and 2D interaction pose (**D**).

Based on the preliminary studies, that include molecular docking, ADME predictions and toxicity calculations, molecule 64 was identified as a potential hit molecule. Molecule 64 and the standard drug donepezil were submitted for MD simulations using the Desmond tool from Schrodinger. The docking output files of both complexes were used to carry out further MD simulations for 100 ns. Analysis of the results for molecule 64, in particular, the RSMD plot, indicated that the complex was stabilized after 80 ns. To further confirm the stability of the complex, the simulation was extended to 200 ns. With molecule 64, the complex was observed to be stabilized by both hydrogen bonding and hydrophobic interactions. From the RMSD plot, it could be determined that the complex underwent fluctuations until 80 ns and was later stabilized until the end of the 200 ns simulation. However, the fluctuations observed were found to be less than 2 A°. Overall, the RMSD plot clearly indicated that the complex was stable, with deviations not more than 2 A°. Using the RMSF plot, fluctuations were observed with the terminal amino acids. The 2D interaction diagram showed that the complex was stabilized by hydrogen bonding with TYR72, PHE295, and TYR124, and pi–pi stacking interactions with TRP286 and TYR341. Interestingly, one water bridge was observed involving TYR72, and the same residue has formed direct hydrogen bonding with the ligand. Intramolecular hydrogen bonding was also observed within the ligand for almost 80% of the simulation time (Figure 8). Further experimental investigation might confirm the significance of water and the interactions. 

**Figure 9:**
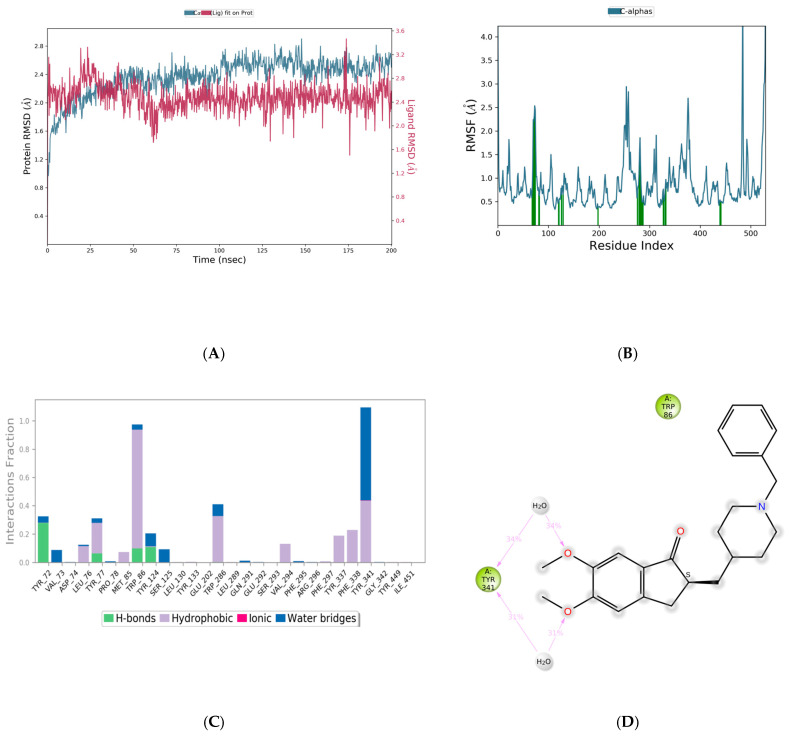
Molecular Dynamics Simulation plots of donepezil (standard) complexed with acetylcholinesterase (PDB ID: 4EY6). RMSD plot (**A**); RMSF plot (**B**); hydrogen bonding interactions hydrogen bonding interactions, note for 8B: Green-Protein residues that interact with the ligand are marked with green-colored vertical bar (**C**); and 2D interaction pose (**D**).

With the standard drug donepezil, the complex was found to be stabilized more through hydrophobic interactions than by hydrogen bonding. From the RMSD plot, it could be seen that the complex equilibrated for the first 10 ns and that later the complex was stable throughout the 200 ns simulation, with only very minor drifts. All the deviations were in the acceptable range of less than 1 A°. Overall, the RMSD plot clearly indicated that the complex was found to be stable, with deviations not more than 1 A°. Using the RMSF plot, fluctuations were observed with terminal amino acids, as with molecule 64. In particular, small fluctuations were observed for residues between 250–400. Interestingly, the binding of molecule 64 had slightly reduced fluctuations in comparison with the standard drug donepezil. The 2D interaction diagram confirmed the hydrogen bonding with TYR341, though mediated through a water bridge. TYR341, an aromatic residue, is a part of the active site that plays a central role in the transfer of the substrate to the binding site. Several studies have reported such interactions between TYR341 and ligand molecules [27,28,29]. Molecule 64 was also found to stabilize through a stacking interaction with the same residue (Figure 9). Overall calculations propose molecule 64 as a hit molecule for further optimization and experimental investigation.

### 3.6. DFT Calculations

HOMO and LUMO energy values play a significant role in strong binding in the active pocket of target proteins and achieving maximum therapeutic benefits. The energy values of hit molecule 64 and the standard drug are recorded in Table 3. Hit molecule 64 had an EHOMO of −7.4600 ev and an ELUMO of −4.6123 ev, with an energy gap of 2.8476 ev. Similarly, donepezil was observed to have an energy gap of 1.6280 ev. These calculations reveal that the hit molecule is more stable than the standard drug.

## 4. Conclusions

The crystal structure of recombinant human acetylcholinesterase in complex with galantamine (PDB ID: 4EY6) was used to generate a structure-based pharmacophore model. The CMNPD library was screened using the generated pharmacophore filter, and compounds that passed through the filter were retrieved from the same database. The selected molecules were then subjected to ADME predictions. Molecular docking analysis of the compounds possessing drug-like properties was performed using AutoDock Tools. The top ten molecules, based upon their docking scores, were selected, and subjected to virtual toxicity testing. Molecule 64 (CMNPD 8741) had the most negligible toxic dose (4000 mg/kg) and was studied using MD simulations. MD studies and DFT calculations confirmed that molecule 64 is stable and confirmed it as a hit compound. According to the ADME studies, this molecule contains a greater number of hydrogen bond donors and acceptors than the standard drug donepezil. The molecule had a similar bioavailability and GI absorption pattern to donepezil, features of great importance in drug design. Molecule 64 exhibited a lower docking score (−11.1 kcal/mol) with AChE than donepezil (−10.4 kcal/mol). Molecular docking studies suggested that the stacked interaction and hydrogen bonding of this molecule with TYR341 made it stable.

Molecule 64 is a bioactive compound known as δ-Indomycinone, that belongs to the family of anthraquinones and is isolated from marine *Streptomyces* sp. δ-Indomycinone is a new member of the pluramycin class of antibiotics [30]. Indomycinones are generally said to possess anticancer, antibacterial and antiviral properties [31].

In vitro toxicity testing and AChE inhibition studies of the hit molecule are required to further confirm its potential application for the treatment of AD. Therefore, in order to carry out in vitro studies in the future, δ-Indomycinone will be chemically synthesized by the Diels-Alder reaction, using bromonaphthoquinone and 1-methoxy-3-methyl-1-trimethylsiloxy-1,3-butadiene as a starting material [31]. The AChE inhibition activity of the chemically synthesized δ-Indomycinone will be assessed by performing Ellman’s colorimetric assay, where the effect of the compound on AChE activity is determined by measuring the intensity of the yellow color that is produced from thiocholine when it reacts with dithiobisnitrobenzoate ions [32]. Any reduction in Aβ aggregation in response to treatment with δ-Indomycinone can be tested using the Thioflavin S (ThS) fluorescence assay. This assay measures changes in the fluorescence intensity of ThS, which depends upon the presence of Aβ [33]. The suggested studies will further support our conclusion from the virtual screening of marine natural compounds, and the AChE inhibition potential of molecule 64 will be confirmed.

This study opens a new avenue and suggests the exploratory potential of Indomycinones for their various pharmacological actions. The pharmacological potential of many such compounds that have already been reported are yet to be discovered. This is possible by performing virtual screening of such compounds reported in available libraries and datasets.

## Figures and Tables

**Table 1:** The docking scores, interacting amino acid residues and molecular interactions of the top ten molecules and a standard AChEI.

Molecule	CMNPD ID	Docking Score	Interacting Amino Acid Residues	Interactions Involved
223	CMNPD24838	−12.6	GLY122, GLY121	Conventional hydrogen bonding
			VAL294, GLU202, GLY448	Carbon hydrogen bonding
			PHE295	Donor–donor interaction
			TRP86, TYR337	Pi–donor hydrogen bond
			TRP286	Pi–sigma interaction
			TRP286	Pi–pi stacked interaction
			TYR341, TYR124, TYR337	Pi–pi T-shaped interaction
			TRP286	Pi–alkyl interaction
34	CMNPD4433	−11.7	TRP86, SER125	Conventional hydrogen bonding
			TYR124	Pi–pi T-shaped interaction
			TRP286	Pi–pi stacked interaction
			TRP286	Pi–sigma interaction
			TRP86	Pi–donor hydrogen bonding
			TYR72, PHE297, TYR341, PHE338, TRP286	Pi–alkyl interaction
306	CMNPD30440	−11.4	GLY122, GLY121	Conventional hydrogen bonding
			HIS447	Carbon hydrogen bonding
			TRP286	Pi–sigma interaction
			TYR124	Pi–lone pair interaction
			TRP286	Pi–pi stacked interaction
			TYR124, TYR337, TRP86	Pi–pi T-shaped interaction
			TYR72, PHE297, PHE338	Pi–alkyl interaction
89	CMNPD13187	−11.3	PHE295, SER293	Conventional hydrogen bonding
			TRP286, TYR341	Pi–pi stacked interaction
151	CMNPD19682	−11.2	TYR341, GLN291, SER293	Conventional hydrogen bonding
			TRP286	Pi–pi stacked interaction
			TRP286	Amide pi-stacked interaction
			PHE338, TYR337	Pi–alkyl interaction
			HIS287	Van der Waal’s interaction
64	CMNPD8741	−11.1	PHE295, SER293, GLN291	Conventional hydrogen bonding
			TRP286	Pi–pi stacked interaction
			TRP286	Amide pi-stacked interaction
			HIS287	Van der Waal’s interaction
			TYR341	Pi–sigma interaction
27	CMNPD3303	−11.0	TRP286	Pi–pi stacked interaction, pi–alkyl interaction
			TYR341	Pi–donor hydrogen bonding
58	CMNPD7644	−10.9	PHE295	Conventional hydrogen bonding
			VAL294	Carbon hydrogen bonding
			TRP286	Pi–pi stacked interaction, pi–alkyl interaction
			TYR341	Pi–pi stacked interaction
208	CMNPD23795	−10.8	PHE295	Conventional hydrogen bonding
			TRP286	Pi–pi stacked interaction
			TYR341	Pi–donor hydrogen bonding
79	CMNPD12415	−10.7	PHE295, SER293	Conventional hydrogen bonding
			TYR341, TRP286	Pi–pi stacked interaction
			TRP286	Pi–donor hydrogen bonding
			TYR337	Pi–alkyl interaction
			TYR341	Pi–sigma interaction
			PHE297	Pi–pi T-shaped interaction
			LEU289	Alkyl interaction
Standard	Donepezil	−10.4	GLY121	Van der Waal’s interaction
			TRP286	Pi–sigma interaction
			TRP286	Pi–pi stacked interaction
			GLY120	Amide pi-stacked interaction
			TYR337, PHE338, TRP286	Pi–alkyl interaction
Standard	Galantamine	−9.1	GLU202	Conventional hydrogen bonding
			TYR337	Carbon hydrogen bonding
			TRP86	Pi–sigma interaction
			GLY121	Amide pi-stacked interaction
			TRP286, PHE338, PHE297, PHE295, GLY122, HIS447	Pi–alkyl interaction
			GLY122	Van der Waal’s interaction

**Table 2:** Toxicity data of the top ten in silico potential AChE inhibitors.

Molecule	Compound ID	Docking Score (kcal/mol)	Molecular Formula	Predicted Toxic Dose (mg/kg)	Toxicity Class
223	CMNPD24838	−12.6	C_26_H_33_NO_7_	3000	5
34	CMNPD4433	−11.7	C_26_H_38_O_6_	3000	5
306	CMNPD30440	−11.4	C_25_H_26_O_7_	832	4
89	CMNPD13187	−11.3	C_22_H_20_O_7_	2000	4
151	CMNPD19682	−11.2	C_23_H_18_O_6_	450	4
64	CMNPD8741	−11.1	C_24_H_22_O_7_	4000	5
27	CMNPD3303	−11.0	C_20_H_20_O_4_	2400	5
58	CMNPD7644	−10.9	C_20_H_13_ClO_5_	2000	4
208	CMNPD23795	−10.8	C_19_H_11_N_3_O_2_	1600	4
79	CMNPD12415	−10.7	C_26_H_28_O_8_	832	4
Standard	Donepezil	−10.4	C_24_H_29_NO_3_	505	4
Standard	Galantamine	−9.1	C_17_H_21_NO_3_	85	3

**Table 3:** Molecular orbital energy calculations of the hit molecule (molecule 64) and the standard drug donepezil.

Compound Name	HOMO	E_HOMO_ (ev)	LUMO	E_LUMO_ (ev)	Energy Gap (Δev)
Molecule 64	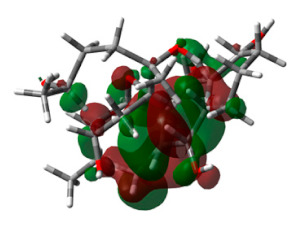	−7.4600	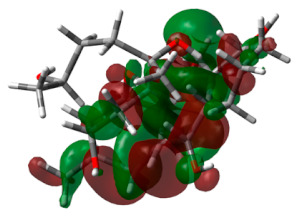	−4.6123	2.8476
Standard	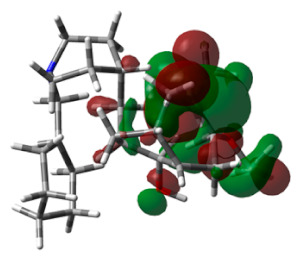	−0.2621	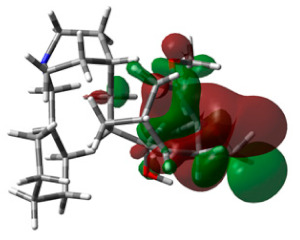	−5.5059	1.6280

## Data Availability

All the data generated during the study is included in manuscript and Supplementary File. No additional data is available to share as research data.

## Supplementary Materials

The following supporting information can be downloaded at: https://www.mdpi.com/article/10.3390/life13061298/s1, Figures S1–S10: Interactions of top 10 molecules with AChE. Table S1: SWISS ADME data.
