# Supplementary material for: Virtual Screening of a Marine Natural Product Database for In Silico Identification of a Potential Acetylcholinesterase Inhibitor
Source: Life (Basel). 2023 May 31;13(6):1298. doi: 10.3390/life13061298 (PMC10301296; doi:10.3390/life13061298)
Supplement: Supplementary file 1 [file life-13-01298-s001.zip › life-2348837-supplementary.pdf]

Figures S1-S10: Interactions of top 10 molecules with AChE

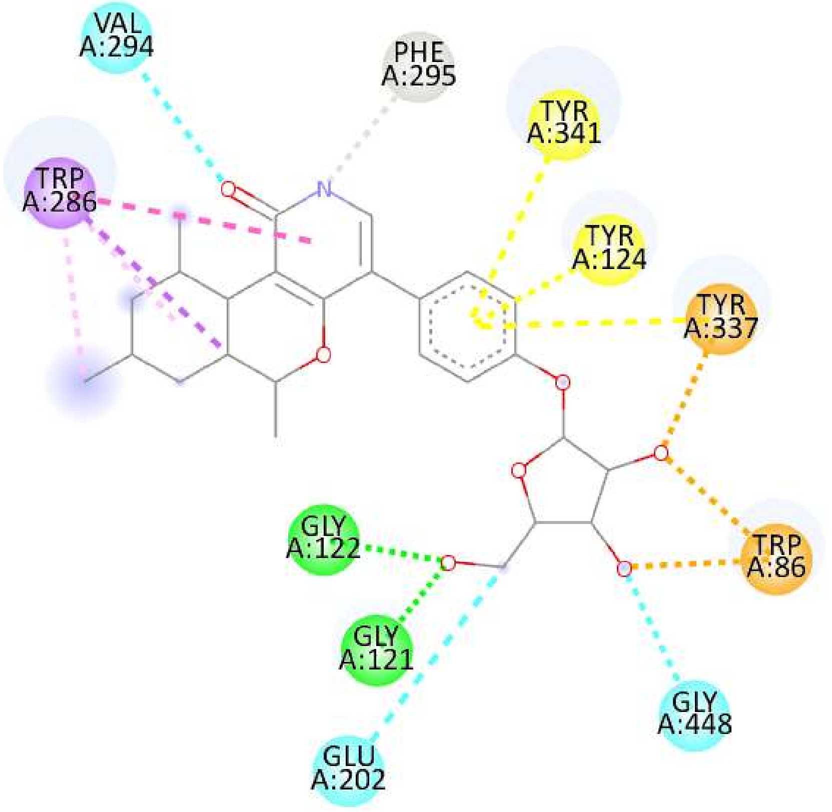

Figure S1: Interactions of CMNPD24838 with target protein

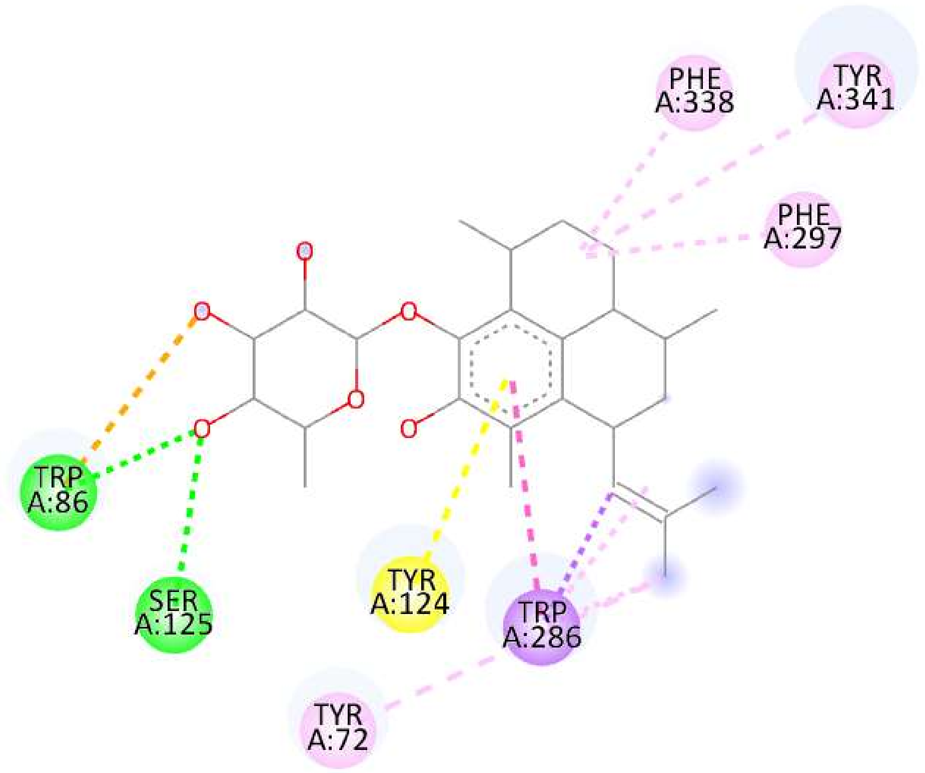

Figure S2: Interactions of CMNPD4433 with target protein

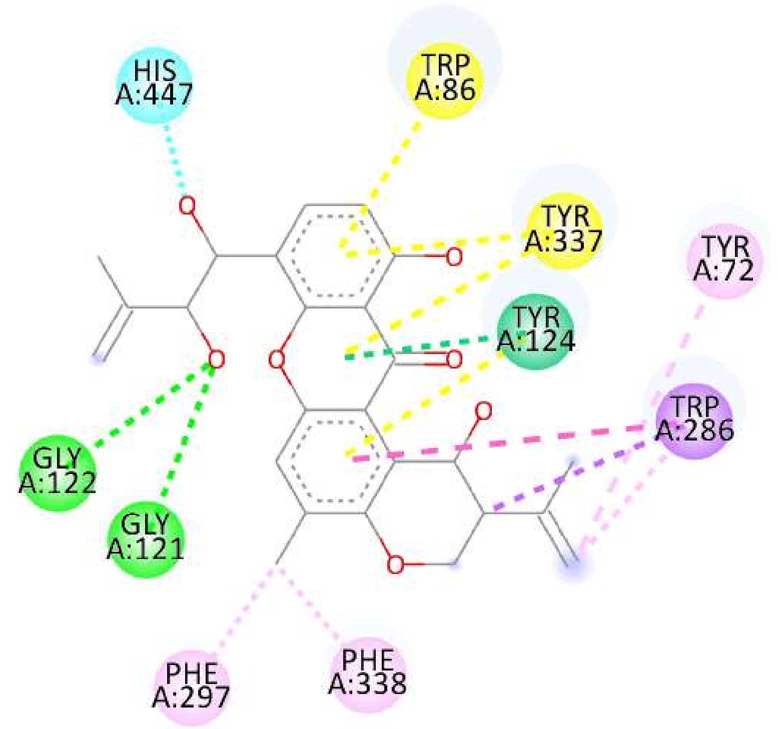

Figure S3: Interactions of CMNPD30440 with target protein

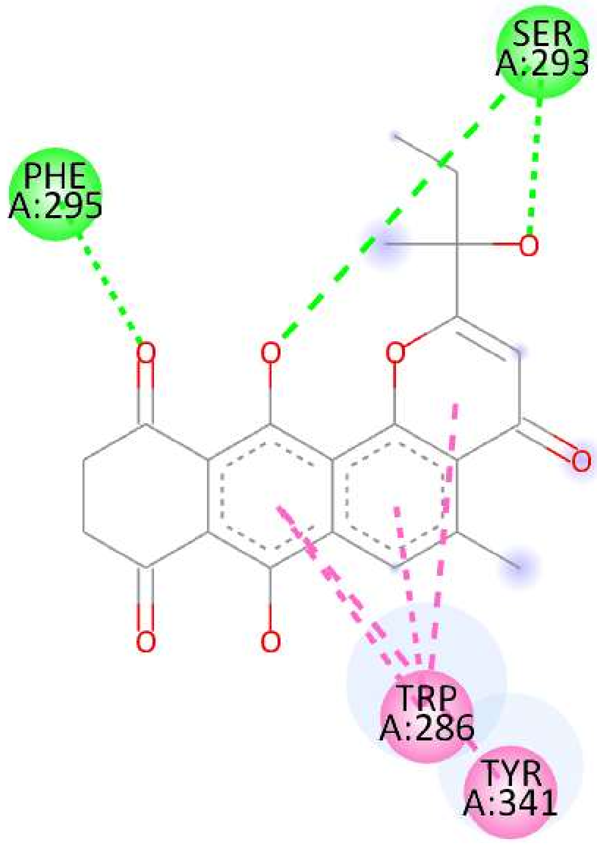

Figure S4: Interactions of CMNPD13187 with target protein

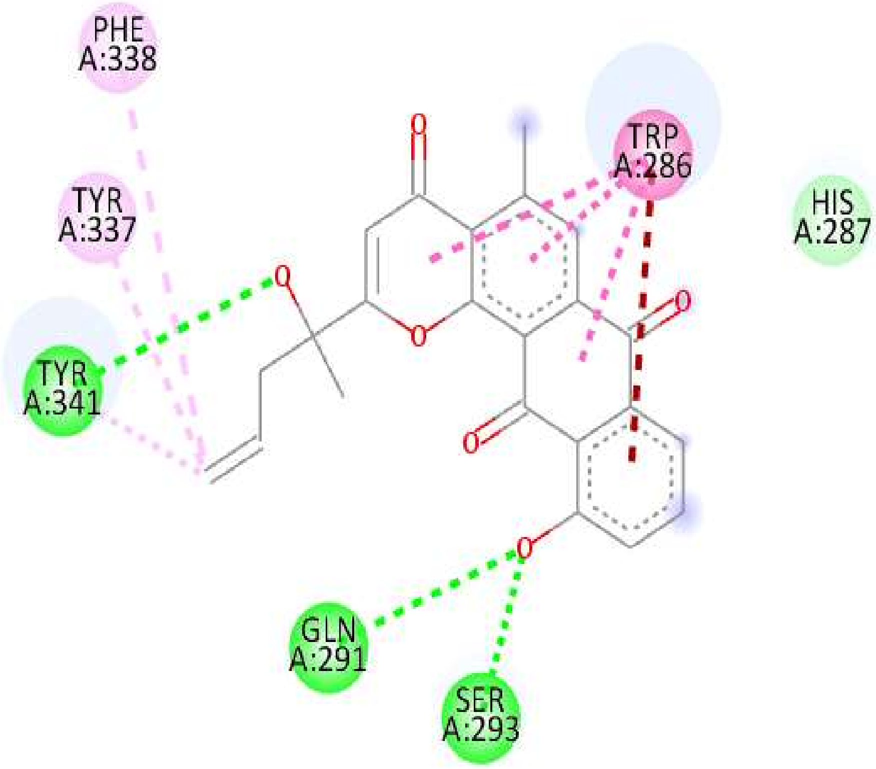

Figure S5: Interactions of CMNPD19682 with target protein

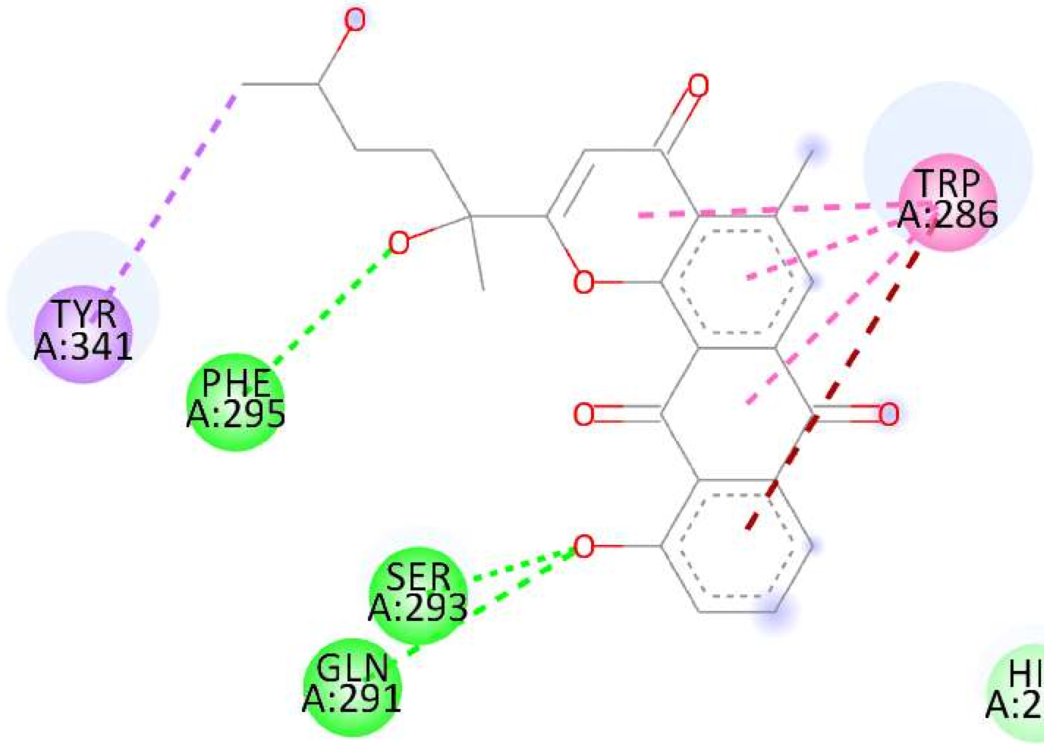

Figure S6: Interactions of CMNPD8741 with target protein

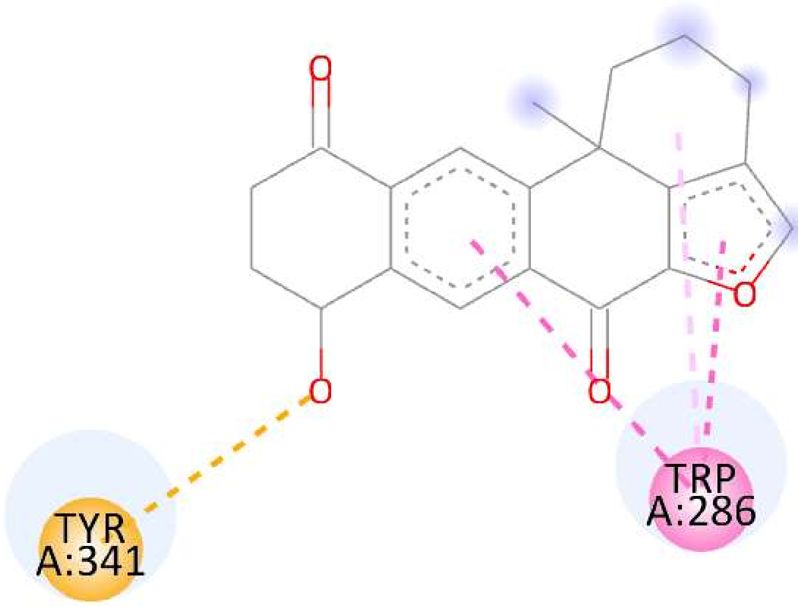

Figure S7: Interactions of CMNPD3303 with target protein

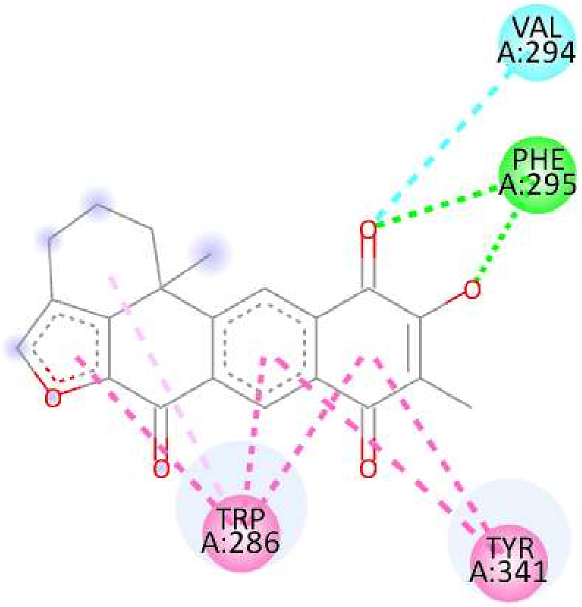

Figure S8: Interactions of CMNPD7644 with target protein

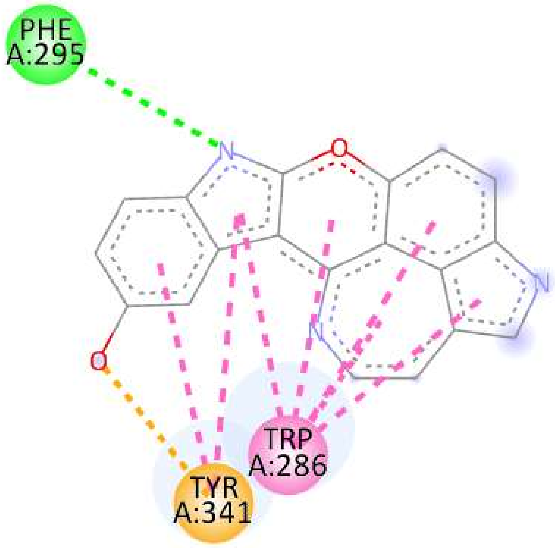

Figure S9: Interactions of CMNPD23795 with target protein

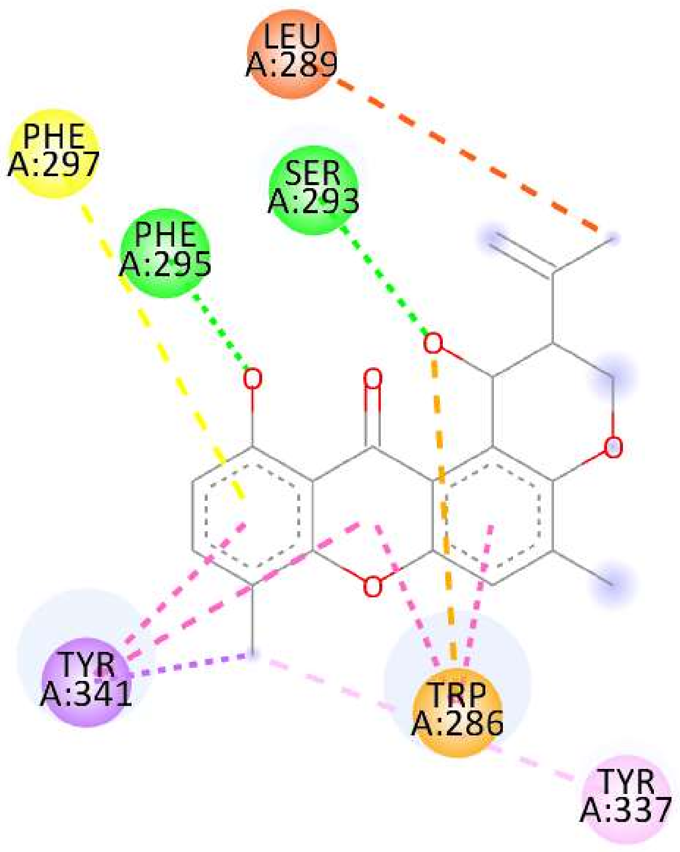

Figure S10: Interactions of CMNPD12415 with target protein

Table S1: SWISS ADME data

Sr. No	Molecular formula	MW	nHA	nAR	nRB	HBA	HBD	MR	TPSA	XLOGP	WLOGP	logP	GI	BBB
1	C14H16BrN3O2S	370.26	21	9	0	4	3	91.15	99.81	2.02	1.79	1.54	High	No
2	C14H16BrN3O2S	370.26	21	9	0	4	3	91.15	99.81	2.02	1.79	1.54	High	No
3	C14H16BrN3O2S	370.26	21	9	0	4	3	91.15	99.81	2.02	1.79	1.54	High	No
4	C14H16BrN3O2S	370.26	21	9	0	4	3	91.15	99.81	2.02	1.79	1.54	High	No
5	C14H16BrN3OS	354.27	20	9	0	3	2	89.13	79.58	2.37	2.09	2.1	High	No
6	C14H16BrN3OS	354.27	20	9	0	3	2	89.13	79.58	2.37	2.09	2.1	High	No
7	C14H16BrN3OS	354.27	20	9	0	3	2	89.13	79.58	2.37	2.09	2.1	High	No
8	C14H16BrN3OS	354.27	20	9	0	3	2	89.13	79.58	2.37	2.09	2.1	High	No
9	C23H24O7	412.43	30	6	5	7	1	109.17	106.97	3.8	3.55	1.07	High	No
10	C25H34O6	430.53	31	6	7	6	1	120.47	89.27	2.89	3.79	1.76	High	No
11	C25H34O6	430.53	31	6	7	6	1	120.47	89.27	2.89	3.79	1.76	High	No
12	C25H34O6	430.53	31	6	7	6	1	120.47	89.27	2.89	3.79	1.76	High	No
13	C25H34O6	430.53	31	6	7	6	1	120.47	89.27	2.89	3.79	1.76	High	No
14	C25H34O6	430.53	31	6	7	6	1	120.47	89.27	2.89	3.79	1.76	High	No
15	C25H34O6	430.53	31	6	7	6	1	120.47	89.27	2.89	3.79	1.76	High	No
16	C25H34O6	430.53	31	6	7	6	1	120.47	89.27	2.89	3.79	1.76	High	No
17	C25H34O6	430.53	31	6	7	6	1	120.47	89.27	2.89	3.79	1.76	High	No
18	C25H34O6	430.53	31	6	7	6	1	120.47	89.27	2.89	3.79	1.76	High	No
19	C25H34O6	430.53	31	6	7	6	1	120.47	89.27	2.89	3.79	1.76	High	No
20	C25H34O6	430.53	31	6	7	6	1	120.47	89.27	2.89	3.79	1.76	High	No
21	C25H34O6	430.53	31	6	7	6	1	120.47	89.27	2.89	3.79	1.76	High	No
22	C25H38O6	434.57	31	6	6	6	4	121.83	99.38	4.63	3.88	2.28	High	No
23	C27H40O7	476.6	34	6	8	7	3	131.57	105.45	5.21	4.45	2.61	High	No
24	C27H40O7	476.6	34	6	8	7	3	131.57	105.45	5.21	4.45	2.61	High	No
25	C20H18O6	354.35	26	6	1	6	2	94	108.74	2.88	3.02	0.13	High	No
26	C13H16N2O4	264.28	19	6	8	5	3	67.4	102.58	-0.56	-0.21	-0.06	High	No
27	C20H20O4	324.37	24	11	0	4	2	88.51	70.67	2.99	2.68	1.7	High	Yes

28	C20H20O4	324.37	24	11	0	4	2	88.51	70.67	2.99	2.68	1.7	High	Yes
29	C22H17NO6S	423.44	30	11	0	6	1	109.73	118.9	3.15	2.77	0.13	High	No
30	C22H17NO6S	423.44	30	11	0	6	1	109.73	118.9	3.15	2.77	0.13	High	No
31	C22H15NO7S	437.42	31	11	0	7	1	110.15	135.97	1.87	2.42	-0.73	High	No
32	C19H34N4S	350.57	24	0	3	4	4	113.42	73.42	1.81	0.24	2.12	High	No
33	C25H36O6	432.55	31	6	3	6	4	119.87	99.38	4.17	3.59	2.28	High	No
34	C26H38O6	446.58	32	6	3	6	4	124.68	99.38	4.6	3.98	2.48	High	No
35	C28H40O7	488.61	35	6	5	7	3	134.42	105.45	5.17	4.55	2.81	High	No
36	C28H40O7	488.61	35	6	5	7	3	134.42	105.45	5.17	4.55	2.81	High	No
37	C26H38O6	446.58	32	6	3	6	4	124.68	99.38	4.6	3.98	2.48	High	No
38	C28H40O7	488.61	35	6	5	7	3	134.42	105.45	5.17	4.55	2.81	High	No
39	C23H29NO6	415.48	30	5	5	7	1	111.72	89.21	3.24	2.62	1.72	High	No
40	C13H16N2O4	264.28	19	6	8	5	3	67.4	102.58	-0.56	-0.21	-0.06	High	No
41	C19H21BrN2O2	389.29	24	15	7	3	3	100.33	57.28	3.39	3.48	2.49	High	Yes
42	C19H20Br2N2O2	468.18	25	15	7	3	3	108.03	57.28	4.08	4.24	3.09	High	Yes
43	C22H26O7	402.44	29	5	5	7	1	105.1	95.2	3.06	2.7	1.51	High	No
44	C21H24O7	388.41	28	5	4	7	2	100.37	106.2	2.52	2.05	1.29	High	No
45	C22H26O7	402.44	29	5	5	7	1	105.1	95.2	3.06	2.7	1.51	High	No
46	C21H24O7	388.41	28	5	4	7	2	100.37	106.2	2.52	2.05	1.29	High	No
47	C20H20O5	340.37	25	6	0	5	2	89.58	83.83	0.98	1.88	0.91	High	No
48	C20H22O5	342.39	25	6	0	5	3	90.32	86.99	0.78	1.41	0.99	High	No
49	C20H22O5	342.39	25	6	0	5	3	90.32	86.99	0.78	1.41	0.99	High	No
50	C20H22O5	342.39	25	6	0	5	3	90.32	86.99	0.78	1.41	0.99	High	No
51	C22H19NO6S	425.45	30	6	0	6	1	112.03	114.99	2.32	3.05	1.25	High	No
52	C17H10O5S	326.32	23	15	2	5	2	86.02	112.07	4.11	3.31	1.79	High	No
53	C22H18O6	378.37	28	16	2	6	2	103.21	104.81	3.29	3.09	0.66	High	No
54	C24H26N5O5+	464.49	34	12	8	7	2	141.83	115.83	1.42	-0.11	1.64	High	No
55	C24H26N5O5+	464.49	34	12	8	7	2	141.83	115.83	1.42	-0.11	1.64	High	No
56	C24H26N5O5+	464.49	34	12	8	7	2	141.83	115.83	1.42	-0.11	1.64	High	No
57	C22H19NO7S	441.45	31	11	4	7	2	109.5	139.13	3.37	3.29	0.16	Low	No
58	C20H13ClO5	368.77	26	11	0	5	1	92.92	84.58	4.43	3.85	0.86	High	No

59	C22H17NO6S	423.44	30	11	0	6	1	109.73	118.9	3.15	2.77	0.13	High	No
60	C10H13NO3S3	291.41	17	6	4	4	1	71.9	131.36	0.92	2.85	0.42	High	No
61	C21H20O7	384.38	28	12	5	7	1	98.06	103.04	1.79	2.07	1.18	High	No
62	C24H22O7	422.43	31	16	4	7	3	113.99	125.04	2.86	2.84	0.28	High	No
63	C24H22O7	422.43	31	16	4	7	3	113.99	125.04	2.86	2.84	0.28	High	No
64	C24H22O7	422.43	31	16	4	7	3	113.99	125.04	2.86	2.84	0.28	High	No
65	C24H22O7	422.43	31	16	4	7	3	113.99	125.04	2.86	2.84	0.28	High	No
66	C24H22O7	422.43	31	16	4	7	3	113.99	125.04	2.86	2.84	0.28	High	No
67	C24H34O5	402.52	29	6	6	5	2	114.44	75.99	5.18	4.5	3.47	High	No
68	C12H9Br2N5O2	415.04	21	15	3	4	4	83.93	111.03	2.92	2.42	1.85	High	No
69	C17H16N3O2+	294.33	22	17	2	3	5	87.67	96.41	1.29	1.66	0.97	High	No
70	C17H16N3O2+	294.33	22	17	2	3	5	87.67	96.41	1.29	1.66	0.97	High	No
71	C16H12O7	316.26	23	12	1	7	5	78.78	135.29	2.06	1.01	-0.73	High	No
72	C17H14O7	330.29	24	12	2	7	4	83.51	124.29	2.05	1.67	-0.49	High	No
73	C17H14O7	330.29	24	12	2	7	4	83.51	124.29	2.05	1.67	-0.49	High	No
74	C14H16BrN3O2S	370.26	21	9	0	4	3	91.15	99.81	2.02	1.79	1.54	High	No
75	C14H16BrN3OS	354.27	20	9	0	3	2	89.13	79.58	2.37	2.09	2.1	High	No
76	C18H22N2O7	378.38	27	9	6	7	3	93.79	133.24	-0.45	-0.46	-0.65	High	No
77	C19H21BrN2O2	389.29	24	15	7	3	3	100.33	57.28	3.39	3.48	2.49	High	Yes
78	C22H21N3O4	391.42	29	15	6	5	3	109.88	107.37	1.59	1.72	0.37	High	No
79	C26H28O8	468.5	34	14	6	8	3	128.11	126.43	4.1	3.11	1.29	High	No
80	C27H24N3O3+	438.5	33	25	4	4	5	128.58	92.39	2.88	2.72	2.09	High	No
81	C27H24N3O3+	438.5	33	25	4	4	5	128.58	92.39	2.88	2.72	2.09	High	No
82	C19H15N3O4	349.34	26	17	0	5	4	100.12	109.68	1.49	1.31	0.17	High	No
83	C19H15N3O4	349.34	26	17	0	5	4	100.12	109.68	1.49	1.31	0.17	High	No
84	C16H10O3S	282.31	20	15	0	3	2	79.71	85.77	4.32	3.83	2.22	High	No
85	C15H11NO6	301.25	22	9	3	6	2	74.19	105.83	0.68	0.7	0.3	High	No
86	C29H28N4O4	496.56	37	20	2	5	2	146.32	80.89	3.35	3.44	2.28	High	No
87	C27H24N4O4	468.5	35	20	1	5	3	136.52	103.67	2.37	2.83	1.89	High	No
88	C27H24N4O4	468.5	35	20	1	5	4	136.69	100.68	2.35	2.44	1.89	High	No
89	C22H20O7	396.39	29	14	2	7	3	107.71	125.04	2.98	3.33	0.16	High	No

90	C14H11Br3O5	498.95	22	12	3	5	5	93.21	101.15	3.61	3.73	2.83	High	No
91	C10H9NO5	223.18	16	6	1	5	3	58.57	99.77	0.43	-0.38	-1.52	High	No
92	C11H11NO6	253.21	18	9	2	6	3	63.45	105.06	2.07	1.18	-0.22	High	No
93	C27H38O7	474.59	34	6	5	7	3	129.61	105.45	4.74	4.16	2.61	High	No
94	C27H38O7	474.59	34	6	5	7	3	129.61	105.45	4.74	4.16	2.61	High	No
95	C25H36O6	432.55	31	6	3	6	4	119.87	99.38	4.17	3.59	2.28	High	No
96	C21H14O7	378.33	28	16	0	7	5	99.97	135.29	3.44	2.48	0.25	High	No
97	C21H14O7	378.33	28	16	0	7	5	99.97	135.29	3.44	2.48	0.25	High	No
98	C20H26N4O3	370.45	27	9	2	6	2	101.27	101.13	3.22	2.91	1.99	High	No
99	C18H16Cl2O6	399.22	26	12	3	6	4	95.11	107.22	1.94	1.88	1.09	High	No
100	C20H20O6	356.37	26	12	5	6	4	98.48	115.06	4.7	3.37	1.32	High	No
101	C22H21N3O4	391.42	29	15	6	5	3	109.88	107.37	1.59	1.72	0.37	High	No
102	C16H12O5	284.26	21	12	1	5	2	75.25	83.83	3.04	2.19	0.61	High	No
103	C16H20O6	308.33	22	6	1	6	3	78.62	96.22	1.52	1.11	0.13	High	No
104	C22H21N3O5	407.42	30	15	10	6	4	108.58	135.44	1.1	1.53	-0.49	High	No
105	C19H23NO5	345.39	25	6	0	5	3	97.09	95.86	1.8	1.67	1.08	High	No
106	C19H23NO5	345.39	25	6	0	5	3	97.09	95.86	1.8	1.67	1.08	High	No
107	C15H16O5	276.28	20	12	4	5	4	74.85	90.15	2.07	2.09	1.18	High	No
108	C18H26O6	338.4	24	6	0	6	4	90.21	107.22	2.99	2.26	1.42	High	No
109	C18H26O6	338.4	24	6	0	6	4	90.21	107.22	2.99	2.26	1.42	High	No
110	C18H26O6	338.4	24	6	0	6	4	90.21	107.22	2.99	2.26	1.42	High	No
111	C18H26O6	338.4	24	6	0	6	4	90.21	107.22	2.99	2.26	1.42	High	No
112	C18H26O6	338.4	24	6	0	6	4	90.21	107.22	2.99	2.26	1.42	High	No
113	C22H25N3O6	427.45	31	6	2	6	3	120.54	119.41	0.63	-1.06	-0.16	High	No
114	C22H25N3O6	427.45	31	6	2	6	3	120.54	119.41	0.63	-1.06	-0.16	High	No
115	C14H15BrN4O3	367.2	22	9	2	3	3	91.84	108.42	0.6	-1.53	0.98	High	No
116	C14H15BrN4O3	367.2	22	9	2	3	3	91.84	108.42	0.6	-1.53	0.98	High	No
117	C14H15BrN4O3	367.2	22	9	2	3	3	91.84	108.42	0.6	-1.53	0.98	High	No
118	C14H15BrN4O3	367.2	22	9	2	3	3	91.84	108.42	0.6	-1.53	0.98	High	No
119	C24H34O6	418.52	30	6	6	6	3	116.46	96.22	5.37	4.21	2.93	High	No
120	C23H23NO7S	457.5	32	6	0	7	2	116.61	135.22	2.06	1.97	0.15	High	No

121	C26H38O6	446.58	32	6	3	6	4	124.68	99.38	4.6	3.98	2.48	High	No
122	C19H22O8	378.37	27	10	5	8	4	95.8	125.68	1.85	0.88	-0.55	High	No
123	C19H22O8	378.37	27	10	5	8	4	95.8	125.68	1.85	0.88	-0.55	High	No
124	C17H14O7	330.29	24	10	2	7	4	84.27	132.13	2.51	1.35	-0.79	High	No
125	C17H14O7	330.29	24	10	2	7	4	84.27	132.13	2.51	1.35	-0.79	High	No
126	C17H14O7	330.29	24	10	2	7	4	84.27	132.13	2.51	1.35	-0.79	High	No
127	C20H20O4	324.37	24	14	3	4	2	96.83	70.67	4.56	3.81	2.23	High	Yes
128	C23H22BrNO6	488.33	31	18	5	7	5	118.5	125.4	2.95	3.21	1.64	High	No
129	C17H24O7	340.37	24	6	5	7	5	86.21	119.61	1.1	0.03	-0.29	High	No
130	C20H16O7	368.34	27	12	0	7	5	92.89	135.29	0.89	0.84	-0.33	High	No
131	C24H30O6	414.49	30	12	1	6	4	116.55	99.38	3.75	4.53	2.03	High	No
132	C24H30O6	414.49	30	12	1	6	4	116.55	99.38	3.75	4.53	2.03	High	No
133	C16H18O6	306.31	22	6	1	6	3	76.93	96.22	1.09	1.2	0.13	High	No
134	C19H25NO5	347.41	25	6	5	5	3	95.14	99.77	2.03	1.73	0.95	High	No
135	C19H25NO5	347.41	25	6	5	5	3	95.14	99.77	2.03	1.73	0.95	High	No
136	C19H25NO5	347.41	25	6	5	5	3	95.14	99.77	2.03	1.73	0.95	High	No
137	C19H25NO5	347.41	25	6	5	5	3	95.14	99.77	1.85	1.73	0.95	High	No
138	C19H25NO5	347.41	25	6	5	5	3	95.14	99.77	1.85	1.73	0.95	High	No
139	C19H25NO5	347.41	25	6	5	5	3	95.14	99.77	1.85	1.73	0.95	High	No
140	C19H24O5	332.39	24	10	9	5	1	94.18	76.74	4.31	3.73	1.91	High	Yes
141	C20H26O5	346.42	25	10	10	5	1	98.99	76.74	4.67	4.12	2.14	High	No
142	C19H24O6	348.39	25	10	9	6	2	95.34	96.97	2.61	2.7	1.1	High	No
143	C19H24O6	348.39	25	10	9	6	2	95.34	96.97	2.61	2.7	1.1	High	No
144	C20H26O6	362.42	26	10	10	6	2	100.15	96.97	2.98	3.09	1.32	High	No
145	C20H26O6	362.42	26	10	10	6	2	100.15	96.97	2.98	3.09	1.32	High	No
146	C18H19Br2NO3	457.16	24	6	1	4	2	104.62	52.93	2.99	2.8	2.58	High	Yes
147	C22H30N2O4S2	450.61	30	12	8	6	2	122.73	116	3.97	3.94	2.68	High	No
148	C23H19ClO7	442.85	31	16	4	7	3	113.98	125.04	2.7	2.73	0.28	High	No
149	C23H18O6	390.39	29	16	3	6	2	108.45	104.81	3.87	3.43	0.8	High	No
150	C23H18O6	390.39	29	16	3	6	2	107.66	104.81	3.64	3.23	0.8	High	No
151	C23H18O6	390.39	29	16	3	6	2	107.54	104.81	3.5	3.26	0.8	High	No

152	C26H29N3O6	479.52	35	6	0	6	3	136.14	117.2	1.9	0.36	1.09	High	No
153	C20H18O4	322.35	24	11	0	4	1	87.76	67.51	3.2	3.15	1.62	High	Yes
154	C20H18O4	322.35	24	11	0	4	1	87.76	67.51	3.2	3.15	1.62	High	Yes
155	C21H20O4	336.38	25	10	1	4	1	94.56	55.76	3.22	3.36	2.11	High	Yes
156	C15H7BrO7	379.12	23	12	1	7	4	80.48	132.13	2.57	2.04	0.39	High	No
157	C21H15N3O4	373.36	28	22	4	6	1	103.87	94.31	2.52	2.94	1.93	High	No
158	C18H16O7	344.32	25	10	1	7	3	87.52	113.29	3.39	1.9	1.68	High	No
159	C18H16O6	328.32	24	10	3	6	2	88.17	93.06	4.22	3.01	1.32	High	No
160	C16H15NO5	301.29	22	6	1	6	3	80.16	107.68	1.37	2.02	-0.39	High	No
161	C12H16O6	256.25	18	6	4	6	4	64.77	111.13	0	-0.28	-0.73	High	No
162	C12H16O6	256.25	18	6	4	6	4	64.77	111.13	0	-0.28	-0.73	High	No
163	C12H16O6	256.25	18	6	4	6	4	64.77	111.13	0	-0.28	-0.73	High	No
164	C12H16O6	256.25	18	6	4	6	4	64.77	111.13	0	-0.28	-0.73	High	No
165	C12H16O6	256.25	18	6	4	6	4	64.77	111.13	0	-0.28	-0.73	High	No
166	C12H16O6	256.25	18	6	4	6	4	64.77	111.13	0	-0.28	-0.73	High	No
167	C12H16O6	256.25	18	6	4	6	4	64.77	111.13	0	-0.28	-0.73	High	No
168	C12H16O6	256.25	18	6	4	6	4	64.77	111.13	0	-0.28	-0.73	High	No
169	C12H16O6	256.25	18	6	4	6	4	64.77	111.13	0	-0.28	-0.73	High	No
170	C12H16O6	256.25	18	6	4	6	4	64.77	111.13	0	-0.28	-0.73	High	No
171	C21H22O7	386.4	28	12	4	7	3	101.81	105.45	2.62	2.67	1.24	High	No
172	C16H24O5	296.36	21	6	5	5	5	81.81	101.15	2.17	1.4	1.06	High	No
173	C16H24O5	296.36	21	6	5	5	5	81.81	101.15	2.17	1.4	1.06	High	No
174	C16H24O5	296.36	21	6	5	5	5	81.81	101.15	2.17	1.4	1.06	High	No
175	C16H24O5	296.36	21	6	5	5	5	81.81	101.15	2.17	1.4	1.06	High	No
176	C22H20O7	396.39	29	12	3	7	3	103.69	121.13	2.55	2.42	0.85	High	No
177	C20H19ClO7	406.81	28	12	5	7	5	103.01	135.29	4.66	3.23	0.7	Low	No
178	C21H21ClO7	420.84	29	12	6	7	4	107.48	124.29	4.99	3.53	0.92	High	No
179	C21H21ClO7	420.84	29	12	6	7	4	107.74	124.29	4.65	3.88	0.92	High	No
180	C22H23ClO7	434.87	30	12	7	7	3	112.21	113.29	4.97	4.18	1.13	High	No
181	C20H17ClO6	388.8	27	12	4	6	4	102.17	115.06	5.15	4.03	1.42	High	No
182	C21H19ClO6	402.82	28	12	5	6	3	106.64	104.06	5.48	4.34	1.64	High	No

183	C22H23BrO7	479.32	30	12	7	7	3	114.9	113.29	5.04	4.29	1.24	High	No
184	C22H23BrO7	479.32	30	12	7	7	3	114.9	113.29	5.04	4.29	1.24	High	No
185	C21H21BrO7	465.29	29	12	6	7	4	110.17	124.29	5.05	3.64	1.03	High	No
186	C21H21BrO7	465.29	29	12	6	7	4	110.17	124.29	5.05	3.64	1.03	High	No
187	C22H24O7	400.42	29	12	7	7	3	107.2	113.29	4.34	3.53	0.65	High	No
188	C18H15ClO8	394.76	27	16	4	8	3	98.47	118.59	3.42	3.26	0.15	High	No
189	C18H15ClO8	394.76	27	16	4	8	3	98.47	118.59	3.42	3.26	0.15	High	No
190	C18H15ClO8	394.76	27	16	4	8	3	98.47	118.59	3.42	3.26	0.15	High	No
191	C16H20O6	308.33	22	6	3	6	3	77.76	96.22	0.35	0.36	-0.14	High	No
192	C13H16O6	268.26	19	6	2	6	3	65.88	96.22	0.8	0.34	0.17	High	No
193	C21H16O5	348.35	26	11	1	5	1	92.52	84.58	3.41	2.76	0.86	High	No
194	C26H23NO6	445.46	33	23	7	6	4	124.37	112.15	3.81	4.13	1.97	High	No
195	C26H23NO6	445.46	33	23	7	6	4	124.37	112.15	3.81	4.13	1.97	High	No
196	C21H26O7	390.43	28	5	3	7	2	100.88	106.2	2.38	2.3	1.37	High	No
197	C28H24N4O5	496.51	37	23	2	6	3	142.04	110.51	2.83	3.02	2.59	High	No
198	C25H27ClO6	458.93	32	6	1	6	3	122.5	104.06	3.23	4.15	1.75	High	No
199	C19H20O8	376.36	27	6	1	8	3	91.9	122.52	1.35	1.29	0.16	High	No
200	C13H14O5	250.25	18	9	4	5	1	65.56	68.9	2.03	2.37	0.81	High	Yes
201	C10H8O4	192.17	14	9	1	4	2	50.45	70.67	1.3	2.05	-0.01	High	Yes
202	C24H31NO7	445.51	32	6	3	7	4	117.93	137.06	1.12	2.04	1.45	High	No
203	C24H31NO7	445.51	32	6	3	7	4	117.93	137.06	1.12	2.04	1.45	High	No
204	C24H31NO7	445.51	32	6	3	7	4	117.93	137.06	1.12	2.04	1.45	High	No
205	C16H20O6	308.33	22	6	1	6	3	78.91	96.22	2.26	2.13	1.34	High	No
206	C19H24O6	348.39	25	10	9	6	2	95.34	96.97	2.61	2.7	1.1	High	No
207	C20H26O6	362.42	26	10	10	6	2	100.15	96.97	2.98	3.09	1.32	High	No
208	C19H11N3O2	313.31	24	23	0	3	3	94.76	77.84	3.93	4.8	2.1	High	No
209	C20H13N3O4	359.33	27	20	1	5	4	104.57	111.38	3.45	3.17	-0.62	High	No
210	C24H35NO4	401.54	29	6	9	4	2	119.15	74.85	3.45	4.25	-0.62	High	No
211	C24H28N2O6	440.49	32	14	10	8	2	120.2	118.84	2.5	3.01	2.03	High	No
212	C18H14O7	342.3	25	14	1	7	2	87.6	98.36	2.55	2.2	0.75	High	No
213	C18H14O7	342.3	25	14	1	7	2	87.6	98.36	2.55	2.2	0.75	High	No

214	C18H14O7	342.3	25	14	1	7	2	87.6	98.36	2.55	2.2	0.75	High	No
215	C18H14O7	342.3	25	14	1	7	2	87.6	98.36	2.55	2.2	0.75	High	No
216	C18H14O7	342.3	25	14	1	7	2	87.6	98.36	2.55	2.2	0.75	High	No
217	C21H18O8	398.36	29	12	1	8	3	99.03	122.52	2.33	1.88	0.05	High	No
218	C21H18O8	398.36	29	12	1	8	3	99.03	122.52	2.33	1.88	0.05	High	No
219	C25H27ClO6	458.93	32	14	4	6	3	126.43	100.13	4.79	4.17	2.14	High	No
220	C25H26O6	422.47	31	14	4	6	3	121.12	100.13	4.82	3.73	1.86	High	No
221	C27H32O7	468.54	34	14	6	7	2	132.25	98.36	4.59	4.24	1.54	High	No
222	C27H30O8	482.52	35	14	6	8	3	132.53	126.43	4.09	3.5	1.49	High	No
223	C26H33NO7	471.54	34	12	4	7	4	126.36	121.24	2.32	2.41	1.46	High	No
224	C18H24O5	320.38	23	6	3	5	3	87.87	86.99	3.35	2.31	1.88	High	No
225	C18H24O5	320.38	23	6	3	5	3	87.87	86.99	3.35	2.31	1.88	High	No
226	C19H28O2	288.42	21	6	8	2	2	92.44	40.46	6.22	4.73	3.98	High	Yes
227	C19H16O5	324.33	24	11	0	5	1	84.04	76.74	2.55	2.54	1.64	High	Yes
228	C17H17N3O3	311.34	23	14	3	5	3	89.28	95.34	1.83	2.23	0.64	High	No
229	C20H21N3O4	367.4	27	10	3	5	3	106.68	102.76	1.17	1.8	0.98	High	No
230	C20H19N3O4	365.38	27	10	3	5	3	106.2	102.76	1.37	1.58	0.91	High	No
231	C20H30O7S	414.51	28	6	6	7	3	108.21	130.75	2.48	2.14	0.67	High	No
232	C25H18O7	430.41	32	18	2	7	4	115.87	132.13	4.09	3.18	0.99	High	No
233	C18H20O7	348.35	25	12	7	7	4	90.3	116.45	2.02	2.23	1.3	High	No
234	C18H20O7	348.35	25	12	7	7	4	90.3	116.45	2.02	2.23	1.3	High	No
235	C18H20O6	332.35	24	10	7	6	2	90.85	96.97	2.01	2.28	0.79	High	No
236	C25H28O6	424.49	31	12	5	6	3	119.99	96.22	5.82	4.55	3.09	High	No
237	C25H28O6	424.49	31	12	5	6	3	119.99	96.22	5.82	4.55	3.09	High	No
238	C21H24O7	388.41	28	12	4	7	3	102.32	105.45	2.3	2.89	1.32	High	No
239	C25H36O6	432.55	31	6	9	6	4	123.1	107.22	2.3	4.33	1.32	High	No
240	C25H36O6	432.55	31	6	9	6	4	123.1	107.22	2.3	4.33	1.32	High	No
241	C19H18O6	342.34	25	10	1	6	2	90.94	93.06	3.31	2.62	0.96	High	No
242	C20H22O6	358.39	26	10	1	6	3	96.37	96.22	3.33	2.54	1.26	High	No
243	C20H22O6	358.39	26	10	1	6	3	96.37	96.22	3.33	2.54	1.26	High	No
244	C18H16O7	344.32	25	10	0	7	3	87.55	113.29	3.84	2.25	2.09	High	No

245	C18H16O7	344.32	25	10	0	7	3	87.55	113.29	3.84	2.25	2.09	High	No
246	C18H18O6	330.33	24	10	1	6	2	87.24	85.22	3.74	2.77	2.15	High	No
247	C16H14O6	302.28	22	10	3	6	3	80.22	96.22	3.62	2.83	1.45	High	No
248	C19H17ClO9	424.79	29	10	2	9	3	99.13	135.66	0.63	1.11	-0.04	High	No
249	C23H26O7	414.45	30	12	2	7	3	111.66	105.45	4.53	4.76	2.56	High	No
250	C24H21NO7S	467.49	33	11	0	7	2	119.11	133.5	1.9	2.11	0.25	High	No
251	C24H21NO7S	467.49	33	11	0	7	2	119.11	133.5	1.9	2.11	0.25	High	No
252	C12H12O5	236.22	17	10	2	5	3	62.27	90.9	1.77	1.13	0.5	High	No
253	C22H30O4	358.47	26	12	7	4	4	106.59	80.92	5.62	4.63	3.37	High	No
254	C15H14O7	306.27	22	10	4	7	3	77.98	113.29	2.17	1.95	0.53	High	No
255	C19H17NO6	355.34	26	10	0	7	3	94.97	116.91	4.03	2.74	0.76	High	No
256	C19H17NO6	355.34	26	10	0	7	3	94.97	116.91	4.03	2.74	0.76	High	No
257	C19H17NO6	355.34	26	10	0	7	3	94.97	116.91	4.03	2.74	0.76	High	No
258	C19H17NO6	355.34	26	10	0	7	3	94.97	116.91	4.03	2.74	0.76	High	No
259	C11H12O6	240.21	17	6	1	6	4	57.16	107.22	-0.25	-0.24	-1.2	High	No
260	C14H16O6	280.27	20	10	4	6	4	73.05	111.13	1.51	0.88	0.22	High	No
261	C14H16O6	280.27	20	10	4	6	4	73.05	111.13	1.51	0.88	0.22	High	No
262	C17H14O5	298.29	22	16	1	5	3	83.92	90.9	3.76	3.19	1.82	High	No
263	C14H10O5	258.23	19	14	0	5	3	71.02	90.9	2.81	2.37	0.57	High	No
264	C14H10O6	274.23	20	14	0	6	4	73.05	111.13	2.46	2.08	0.02	High	No
265	C11H10O4	206.19	15	9	2	4	1	54.92	59.67	1.62	2.35	0.28	High	Yes
266	C20H18O8	386.35	28	12	5	8	3	98.61	130.36	3.36	2.58	-0.39	High	No
267	C18H13BrO8	437.2	27	12	3	8	3	96.7	130.36	3.22	2.56	-0.23	High	No
268	C20H17BrO8	465.25	29	12	5	8	3	106.31	130.36	4.05	3.34	0.21	High	No
269	C19H15BrO7	435.22	27	12	4	7	3	99.82	121.13	4.08	3.33	0.51	High	No
270	C24H21NO7S	467.49	33	11	0	7	2	119.11	133.5	1.9	2.11	0.25	High	No
271	C23H30O8	434.48	31	5	6	8	1	112.73	112.27	2.06	2.77	0.94	High	No
272	C17H12O7	328.27	24	12	2	7	3	82.5	121.13	2.56	1.79	-0.56	High	No
273	C22H19NO5	377.39	28	16	3	5	2	107.2	79.23	2.86	2.41	2.15	High	No
274	C18H14N2O5	338.31	25	15	0	6	5	88.76	126.67	0.91	1.37	0.22	High	No
275	C19H17ClO7	392.79	27	16	5	7	1	100.92	87.36	3.55	3.85	0.9	High	No

276	C19H17ClO7	392.79	27	16	5	7	1	100.92	87.36	3.55	3.85	0.9	High	No
277	C19H17ClO7	392.79	27	16	5	7	1	100.92	87.36	3.55	3.85	0.9	High	No
278	C19H18O7	358.34	26	16	5	7	1	95.91	87.36	2.92	3.2	0.4	High	No
279	C19H18O7	358.34	26	16	5	7	1	95.91	87.36	2.92	3.2	0.4	High	No
280	C19H18O7	358.34	26	16	5	7	1	95.91	87.36	2.92	3.2	0.4	High	No
281	C18H20O7	348.35	25	12	7	7	4	90.15	116.45	2.57	2.02	1.3	High	No
282	C15H18O6	294.3	21	10	4	6	4	78.01	111.13	1.87	1.19	0.48	High	No
283	C25H36O7	448.55	32	6	7	7	4	122.59	116.45	4.24	3.54	2.07	High	No
284	C25H36O7	448.55	32	6	7	7	4	122.59	116.45	4.24	3.54	2.07	High	No
285	C21H26O9	422.43	30	12	6	9	5	105.55	138.07	1.41	0.99	-0.3	High	No
286	C28H38N2O5	482.61	35	6	7	5	3	144.44	99.1	4.28	3.24	2.51	High	No
287	C27H37NO7	487.59	35	6	9	7	4	137.76	127.53	3.79	3.18	1.93	High	No
288	C25H29NO5	423.5	31	12	4	5	3	123.09	88.02	4.67	3.25	2.49	High	No
289	C25H29NO5	423.5	31	12	4	5	3	123.09	88.02	4.67	3.25	2.49	High	No
290	C25H28O7	440.49	32	12	6	7	3	120.48	113.29	4.99	4.05	2.02	High	No
291	C17H20O3	272.34	20	11	4	3	2	80.98	53.6	4.29	3.81	2.12	High	Yes
292	C24H34O5	402.52	29	6	6	5	2	114.44	75.99	5.18	4.5	3.47	High	No
293	C25H35NO6	445.55	32	6	7	6	4	124.28	116.09	5.14	3.97	2.66	High	No
294	C19H16O6	340.33	25	12	1	6	3	88.81	104.06	1.26	1.51	0.24	High	No
295	C19H16O6	340.33	25	12	1	6	3	88.81	104.06	1.26	1.51	0.24	High	No
296	C19H18O6	342.34	25	12	1	6	4	89.55	107.22	0.92	1.04	0.32	High	No
297	C19H18O5	326.34	24	12	1	5	3	88.39	86.99	1.9	2.07	1.14	High	No
298	C19H18O7	358.34	26	12	1	7	5	90.75	127.45	-0.3	0.16	-0.48	High	No
299	C19H16O6	340.33	25	12	1	6	3	87.4	99.52	0.69	1.2	0.32	High	No
300	C26H21N3O5	455.46	34	20	0	5	4	130.24	108.88	2.09	2.21	2.09	High	No
301	C25H28N2O6	452.5	33	12	10	6	5	126.21	135.96	2.89	1.79	1.43	High	No
302	C25H28N2O6	452.5	33	12	10	6	5	126.21	135.96	2.89	1.79	1.43	High	No
303	C20H18O7	370.35	27	12	1	7	4	95.81	124.29	2.96	2.59	0.21	High	No
304	C23H26O6	398.45	29	14	2	6	3	113.47	100.13	4.64	4.34	1.86	High	No
305	C23H26O6	398.45	29	14	2	6	3	113.47	100.13	4.64	4.34	1.86	High	No
306	C25H26O7	438.47	32	14	4	7	4	122.28	120.36	3.71	2.9	1.06	High	No

307	C25H26O7	438.47	32	14	4	7	4	122.28	120.36	3.71	2.9	1.06	High	No
308	C18H16O7	344.32	25	12	4	7	5	87.53	135.29	1.39	1.04	-0.52	High	No
309	C18H16O7	344.32	25	12	4	7	5	87.53	135.29	1.39	1.04	-0.52	High	No
310	C18H16O7	344.32	25	12	4	7	5	87.53	135.29	1.39	1.04	-0.52	High	No
311	C18H16O7	344.32	25	12	4	7	5	87.53	135.29	1.39	1.04	-0.52	High	No
312	C25H34O7	446.53	32	6	4	7	3	119.17	113.29	3.62	2.99	1.8	High	No
313	C25H34O7	446.53	32	6	4	7	3	119.17	113.29	3.62	2.99	1.8	High	No
314	C12H12O6	252.22	18	10	3	6	3	63.1	100.13	0.09	0.21	-0.31	High	No
315	C12H10O7	266.2	19	10	3	7	3	63.71	117.2	0.54	0.3	-0.38	High	No
316	C19H18Cl2O8	445.25	29	12	8	8	2	105.85	111.52	4.96	4.48	2.73	High	No
317	C19H19ClO8	410.8	28	12	8	8	1	100.5	100.52	4.29	3.74	2.24	High	No
318	C16H10Cl2O7	385.15	25	14	2	7	3	92.32	117.2	4.47	3.46	1.48	High	No
319	C17H16O5	300.31	22	10	0	5	2	81.57	75.99	4.18	3.15	2.32	High	Yes
320	C17H16O5	300.31	22	10	0	5	2	81.57	75.99	4.18	3.15	2.32	High	Yes
321	C16H20O7	324.33	23	6	4	7	2	79.63	94.45	2.12	1.57	0.81	High	No
322	C16H10O7	314.25	23	12	1	7	4	78.04	132.13	2.23	1.49	-0.81	High	No
323	C25H30O6	426.5	31	12	4	6	3	118.04	96.22	3.6	3.5	1.89	High	No
324	C25H30O6	426.5	31	12	4	6	3	118.04	96.22	3.6	3.5	1.89	High	No
325	C25H28O6	424.49	31	12	4	6	2	117.45	85.22	5.25	4.06	2.9	High	No
326	C26H31NO6	453.53	33	12	5	6	3	128.86	97.25	4.44	3.22	2.84	High	No
327	C26H30O7	454.51	33	12	7	7	2	124.8	102.29	5.32	4.14	2.23	High	No
328	C12H12O6	252.22	18	10	2	6	4	64.29	111.13	1.41	0.83	-0.04	High	No
329	C13H12Cl2O6	335.14	21	10	4	6	3	78.36	100.13	2.72	1.92	0.75	High	No
330	C17H14O7	330.29	24	12	2	7	5	83.58	135.29	2.56	1.21	-0.49	High	No

MW- Molecular weight, **nHA-** number of heavy atoms, **nAR-** number of aromatic heavy atoms, **nRB-** number of rotatable bonds, **HBA-** Hydrogen bond acceptor, **HBD-** Hydrogen bond donor, **TPSA-** Total polar surface area, **MR-** molar refractivity, **nV-** number of violations, **GI-** GI permeation, **BBB-** BBB permeation, (Y-Yes; N-No),
